# Autoregulatory mechanism of enzyme activity by the nuclear localization signal of lysine-specific demethylase 1

**DOI:** 10.1016/j.jbc.2024.107607

**Published:** 2024-07-30

**Authors:** Dulmi Senanayaka, Danyun Zeng, Sahar Alishiri, William J. Martin, Khadijah I. Moore, Roshni Patel, Zigmund Luka, Alexander Hirschi, Nicholas J. Reiter

**Affiliations:** 1 Department of Chemistry, Marquette University, Milwaukee, Wisconsin, USA; 2 Center for Structural Biology, Vanderbilt University School of Medicine, Nashville, Tennessee, USA; 3 Department of Biochemistry, Vanderbilt University School of Medicine, Nashville, Tennessee, USA

**Keywords:** intrinsically disordered protein, nuclear localization signal (NLS), structural biology, histone demethylase, nucleosome, inhibition mechanism, NMR, autoinhibition

## Abstract

The N-terminal region of the human lysine-specific demethylase 1 (LSD1) has no predicted structural elements, contains a nuclear localization signal (NLS), undergoes multiple posttranslational modifications (PTMs), and acts as a protein-protein interaction hub. This intrinsically disordered region (IDR) extends from core LSD1 structure, resides atop the catalytic active site, and is known to be dispensable for catalysis. Here, we show differential nucleosome binding between the full-length and an N terminus deleted LSD1 and identify that a conserved NLS and PTM containing element of the N terminus contains an alpha helical structure, and that this conserved element impacts demethylation. Enzyme assays reveal that LSD1’s own electropositive NLS amino acids 107 to 120 inhibit demethylation activity on a model histone 3 lysine 4 dimethyl (H3K4me2) peptide (K_i_^app^ ∼ 3.3 μM) and histone 3 lysine 4 dimethyl nucleosome substrates (IC_50_ ∼ 30.4 μM), likely mimicking the histone H3 tail. Further, when the identical, inhibitory NLS region contains phosphomimetic modifications, inhibition is partially relieved. Based upon these results and biophysical data, a regulatory mechanism for the LSD1-catalyzed demethylation reaction is proposed whereby NLS-mediated autoinhibition can occur through electrostatic interactions, and be partially relieved through phosphorylation that occurs proximal to the NLS. Taken together, the results highlight a dynamic and synergistic role for PTMs, intrinsically disordered regions, and structured regions near LSD1 active site and introduces the notion that phosphorylated mediated NLS regions can function to fine-tune chromatin modifying enzyme activity.

Gene activation or inactivation is influenced by the extent of histone methylation, which involves the enzymatic addition or removal of a methyl group at specific locations within the N-terminal tails of histone proteins. Methylation occurs at intrinsically disordered regions of histones and the presence or absence of these posttranslational modifications (PTMs) can dramatically alter the properties of nucleosomes and chromatin accessibility. Chromatin-modifying methyltransferase or demethylase enzymes act to install or remove these methyl marks and, much like nucleosomes, are themselves composed of both higher order structure and regions of intrinsic disorder. The reaction chemistry and molecular recognition properties of nucleosome-associated proteins strongly rely on structural plasticity for substrate recognition and active site formation, resulting in methylation patterns that contribute to the dynamic state of chromatin and subsequently alter gene regulation, genomic stability, and cell migration (1, 2, 3, 4).

The lysine-specific histone demethylase 1A (LSD1 or KDM1A) can catalyze the removal of monomethyl and dimethyl functional groups from lysine 4 or lysine 9 of histone H3 (H3K4me1/2 or H3K9me1/2), resulting in repressive or activating transcriptional marks, respectively (5, 6, 7, 8, 9, 10, 11, 12). LSD1 contains structured and intrinsically disordered regions, allowing it to clamp onto the nucleosome and interact with a variety of components, including over 60 distinct proteins and transcription factors, numerous nucleic acid structures, and essential nutrients (1, 2, 13, 14, 15, 16, 17, 18). LSD1 fully engages with nucleosomes upon formation of a stable complex with the C-terminal region of the corepressor for repressor element 1 silencing transcription factor (CoREST) (RCOR1), a key silencing transcription factor. The crystal structure of LSD1-CoREST in complex with the nucleosome revealed the relative modes of nucleosome recognition, the importance of CoREST, and how LSD1 interacts with the nucleosome to achieve optimal demethylase activity (19). In addition to the defined structural elements, the N- terminal locations implying disorder can be gleaned from the LSD1-CoREST-nucleosome complex. Specifically, three regions that include: the N-terminus of LSD1 (aa 1–170, *h. sapiens* numbering), a region of CoREST (aa 280–308), and the H3 tail (aa 15–38) are disordered and absent in the structure. Many of these intrinsically disordered regions (IDRs) represent stretches of positively and negatively charged amino acids and yet each of these disordered regions are poised to cluster in proximity near the active site cleft, positioned 7 to 25 Å from each other and the catalytic pocket. This suggests that flexible, disordered regions may play a dynamic role in protein-protein interactions, nucleosome docking, or in fine-tuning the structural mechanism of activation, as previously shown in other chromatin-associated protein systems (20, 21, 22). The N terminus region of LSD1 (aa 1–170) is dispensable for catalysis *in vitro* yet contains regions of high-sequence conservation among mammals and participates in a variety of regulatory functions (Fig. 1*A*). A nuclear localization signal (NLS) (aa 112–117, RRKRAK) allows for internalization of LSD1 into the nucleus and contains context specific and functionally important PTMs involved in estrogen-responsive or androgen-dependent transcription regulation (23, 24, 25). Many PTMs, such as phosphorylation (aa 110, 111, 131, and 137) and methylation (aa 114,126), influence transcription networks, DNA damage repair mechanisms, and promote the release of LSD1 from chromatin during mitosis (26). In addition, mass spectroscopy analysis of a bifunctional ternary CoREST complex identified high confidence cross-links of LSD1 (aa 117, 144) with both histone deacetylase 1 (HDAC1 aa 89, 123) and CoREST regions (aa 298, 300, and 356) (27). Taken together, these studies identify an extensive protein interaction hub that resides between residues 110 to 145 of human LSD1.

**Figure 1:**
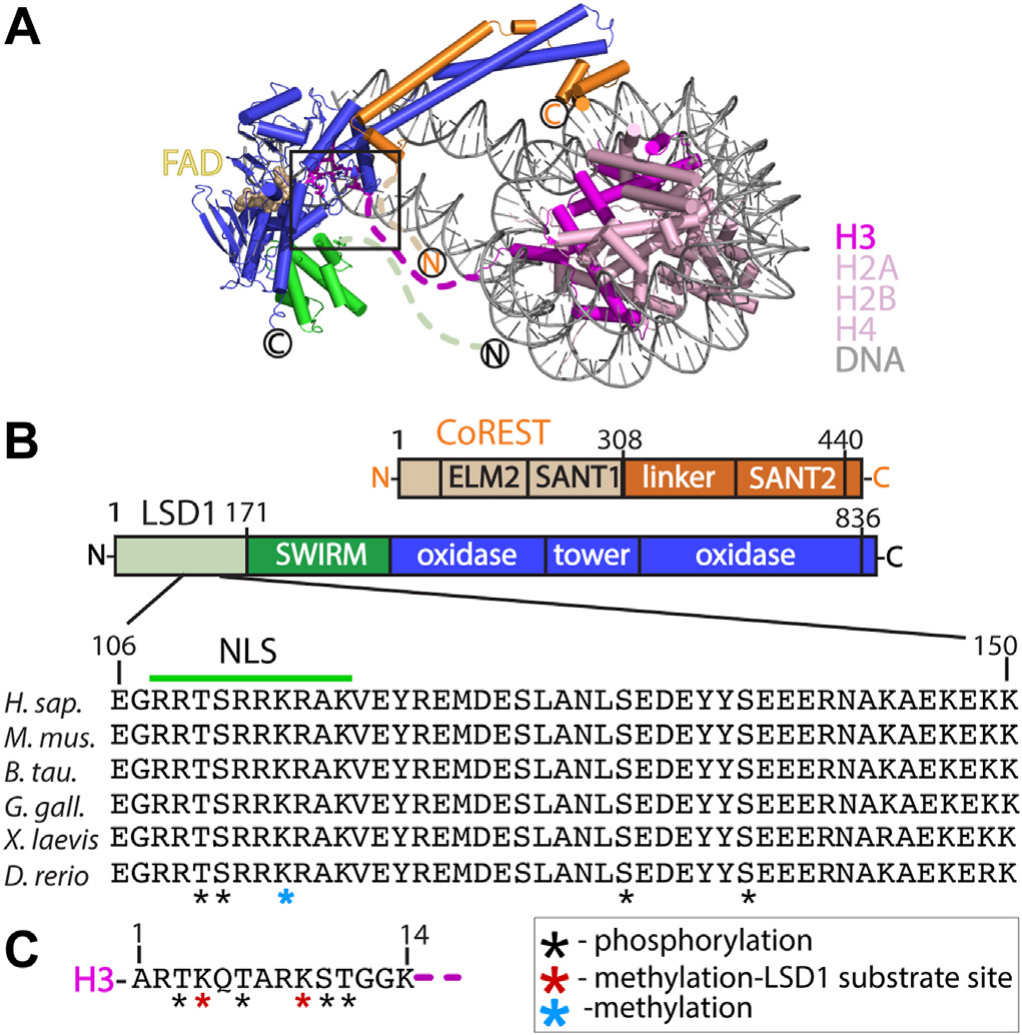
**Structure of the LSD1-CoREST-nucleosome complex, emphasizing disordered regions that reside near the active site cleft.***A*, the LSD1-CoREST-nucleosome structure (PDB 6VYP). Within the *black box* region, the H3 histone tail (*purple dash*), 170 residue N-terminal tail extends from the SWIRM domain (*green dash*) of LSD1, the C terminus extends from CoREST (*orange dash*), and extranucleosomal DNA (*gray*) all reside within 4 to 18 Å from LSD1’s catalytic pocket. Interactions between LSD1 (*blue/green*), CoREST (*orange*), histones (*pink*), and DNA (*gray*) are required for H3K4me2 demethylation. *B*, schematic of CoREST and LSD1, and sequence conservation of an N terminus region spanning residues 106 to 150 of LSD1. Phosphorylation-methylation modifications (*black/blue asterisks*) and residues encompassing the nuclear localization signal (NLS (*green*), 112 to 117) in this region of LSD1 are noted. *C*, the H3 tail sequence can include phosphorylation (*black*) and methyl modifications (*red asterisks*). CoREST, corepressor for repressor element 1 silencing transcription factor; H3K4me2, histone 3 lysine 4 dimethyl; LSD, lysine-specific demethylase; NLS, nuclear localization signal; PDB, Protein Data Bank; SWIRM, The Swi3p, Rsc8p and Moira protein domains.

The extent of protein-protein interactions along with the proximity of disordered residues near the catalytic core suggest the presence of dynamic, tunable elements that may reside within the nucleosome-demethylase interface of LSD1. While this manuscript was under review, colleagues identified that the N terminus IDR of LSD1 undergoes “closed” and “open” conformational equilibrium states to self-modulate LSD1-protein interaction networks, helping to control enhancer silencing mechanisms in the cell (28). Furthermore, it was demonstrated that disordered N-terminal sequences of other intrinsically disordered protein containing transcription factors, such as SNAIL1, INSM1, and GLF1B, possess conserved patterns of positively charged residues that mimic the H3 substrate tail and inhibit the demethylation reaction through direct interactions at LSD1’s catalytic pocket (29, 30). Is there a role for the noncatalytic N-terminus region of LSD1 in H3K4me2 nucleosome demethylation? Here, we demonstrate an unexpected result whereby LSD1’s own conserved NLS region can function as a reversible, competitive inhibitor of demethylation, acting in an autoinhibitory manner. We show that a conserved 45 amino acid IDR within LSD1’s N terminus forms a transient α-helix (residues 135–148) and that the NLS region (residues 107–120) inhibits demethylation on H3K4me2 nucleosome substrates. Further, data reveal that inhibition can be relieved through the incorporation of phosphomimetic mutations. These experiments, along with comparative molecular dynamics (MD) simulations, suggest that LSD1’s own NLS can interact at the active site cleft, contributing to electrostatic-based inhibition. This study highlights the interplay of disorder and structure near enzyme active sites and expands the regulatory role(s) of disordered regions in the immediate vicinity of LSD1’s catalytic pocket. The work also provides insight into the development of PTM-based peptidomimetic inhibitors that may target LSD1, introducing a new example for how a phosphoregulatory NLS region can modulate the function of a chromatin-modifying enzyme.

## Results

### The NLS region at LSD1’s N-terminus is conserved

The N terminus of LSD1 was previously found to have little sequence conservation among the FAD-dependent monoamine oxidase enzyme family (5, 31, 32), yet a significant level of sequence conservation is concentrated within the N-terminus of LSD1 homologs (residues 100–151) in metazoans. Specifically, a 21 amino acid stretch adjacent to the NLS of LSD1 has over 95% sequence identity among vertebrates (Fig. 1*B*). This suggests that the N-terminal region (residues 100–151, herein termed NT-LSD1) contains recognition signatures that play functional roles in the dynamic LSD1 interaction network.

### The N terminus enhances LSD1 binding to nucleosomes

**Figure 2:**
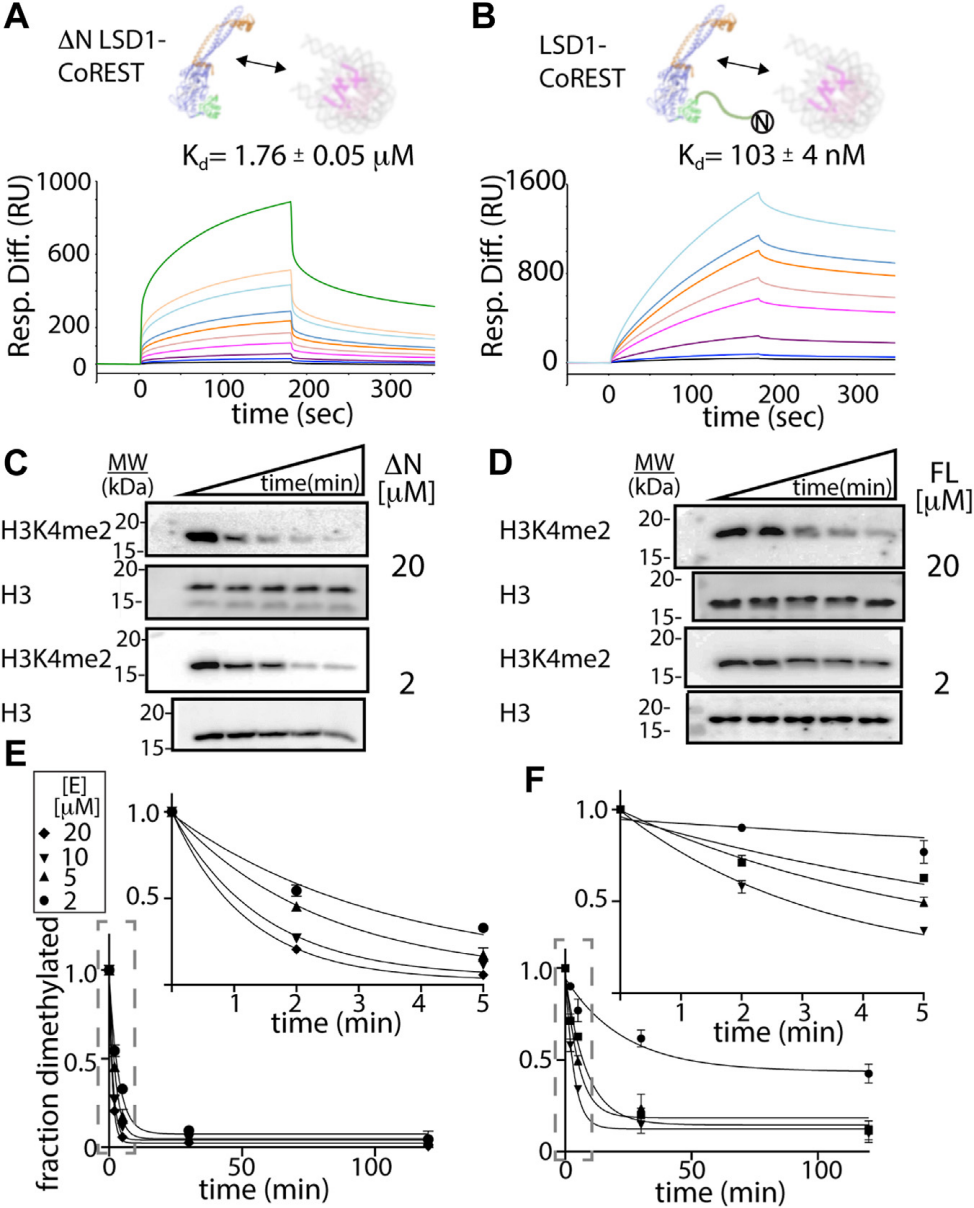
**Comparative binding and activity on nucleosomes by ΔN (171–852) and full-length LSD1.** SPR titration series of (*A*) ΔN LSD1 (171–852)–CoREST (286–482) and (*B*) full-length LSD1 (1–852)-CoREST (286–482) to 147 bp mononucleosomes. Sensorgram data show differential response units (RU) over time, measuring binding to mononucleosomes immobilized *via* biotinylated DNA on a sensor chip. In (*A*-*B*), nucleosomes were subject to 5 (*dark blue*), 10 (*blue*) 2 (*dark red*), 50 (*magenta*), 100 (*pink*), 200 (*orange*), 300 (*turquoise*), 400 (*cyan*), 500 (*light orange*), and 2000 nM (*green*) purified LSD1-CoREST. Differences in the response units (RU) *versus* protein concentration and fitted data (Fig. S2) revealed the apparent binding dissociation constants (K_d_). Western blot activity assays show H3K4me2 nucleosome demethylation by (*C*) ΔN LSD1 (ΔN, 171–852)–CoREST (286–482) and (*D*) full-length LSD1 (FL, 1–852) in complex with CoREST (286–482). Reactions with enzyme (20 and 2 μM, see Fig. S4 for all blots) and in buffer 50 mM Hepes (pH 8.0), 50 mM KCl, 5% glycerol, 1 mM Tris(2-carboxyethyl)phosphine were initiated with 100 nM nucleosome substrate. The degree of inhibition was measured using H3K4me2 specific antibody relative to the amount of H3 in each lane using H3 antibody. *E*-*F*, quantitation of the H3K4me2 antibody signal relative to the H3 antibody signal at enzyme concentration (2, 5, 10, and 20 μM) over time enabled an analysis of LSD1’s demethylation activity. *Image disclosure*: Fig. S3*A* (20 and 2 μM experiments) are redundant with Figs. 2*C* and S3*B* (20 and 2 μM experiments) are redundant with Fig.ure 2*D*. Thus, the same data were used to produce both figures. *Image justification*: images are shown together in the context of the entire Western blot assay series (N = 2). Analysis of the data and all time course experiments enabled the quantification of LSD1 activity on H3K4me2 nucleosome substrates *in vitro*. CoREST, corepressor for repressor element 1 silencing transcription factor; H3K4me2, histone 3 lysine 4 dimethyl; LSD, lysine-specific demethylase; SPR, surface plasmon resonance.

To obtain quantitative interaction data at thermodynamic equilibrium, surface plasmon resonance (SPR) studies were performed comparing binding of the truncated (ΔN, residues 171–852) and full-length (FL, residues 1–852) LSD1 to the nucleosome core particle (Fig. 2, *A* and *B*). Titration experiments using high and low surface densities of unmodified nucleosomes immobilized *via* biotinylated “601” DNA sequence on a sensor chip were measured and analyzed for either ΔN LSD1-CoREST or FL LSD1-CoREST proteins, where LSD1 constructs were coexpressed and purified with the identical C-terminal domain construct of CoREST1 (residues 286–482) (Fig. S1). Sensorgram data of the differential response units (RU) over time show a consistent binding kinetics trend, with 1:1 stoichiometry based on a Langmuir binding model and no detectable cooperative binding for either ΔN LSD1-CoREST or FL LSD1-CoREST (Fig. S2). Analysis of the difference in RU *versus* protein concentration reveal that FL LSD1-CoREST has ∼8 to 12 fold higher affinity toward nucleosomes compared to ΔN LSD1-CoREST in 50 mM KCl salt conditions (Fig. 2, *A* and *B*). Apparent binding dissociation constants (K_d_) of approximately 1760 ± 50 nM and 103 ± 4 nM were obtained for ΔN and FL LSD1-CoREST, respectively, demonstrating that the N-terminal region of LSD1 plays a role to enhance nucleosome binding. Here, it is worth noting that nucleosomes in this study contain only a 147 bp DNA, whereas the optimal DNA length for LSD1-CoREST engagement requires an extended, extranucleosomal > 160 bp DNA (33). Nonetheless, a comparative SPR analysis reveals that the N-terminal residues 1 to 170 enhance the LSD1-nucleosome binding interaction by over an order of magnitude.

### The N terminus impedes LSD1-catayzed demethylation on H3K4me2 nucleosomes

Kinetic measurements of demethylation were also performed under single-turnover conditions by quantifying *in vitro* based Western blots (WBs) from a reaction solution containing 100 nM H3K4me2 nucleosomes. Here, time-course assays measure the fraction of dimethylated H3K4me2 nucleosomes and were monitored using an anti-H3K4me2 specific antibody (Figs. 2, *C* and *D* and S2). The degree of inhibition was measured using H3K4me2-specific antibody relative to the amount of H3 in each lane using H3-specific antibody (Fig. S3). Half of the time-course reaction mixture was subsequently incubated with an H3K4me2-specific antibody on one blot and the other half of the reaction mixture was incubated with an H3 specific antibody in a separate blot. In all cases, the corresponding blots were incubated with either H3K4me2 or H3-specific primary antibodies, and the blot images were visualized by chemiluminescence. Dividing the H3K4me2 antibody signal by the H3 antibody signal allowed for quantitation of each data point, where the disappearance of H3K4me2 signal directly corresponds to LSD1’s activity on nucleosomes.

Analysis of demethylation data on H3K4me2 nucleosomes reveal that ΔN LSD1-CoREST has a k_max_/K_1/2_ of 0.112 ± 0.005 min^−1^ μM whereas the FL LSD1-CoREST has a k_max_/K_1/2_ of 0.034 ± 0.008 min^−1^ μM. Thus, the N-terminal tail sequence noticeably decreases the enzyme’s catalytic efficiency by 3.5 times compared to the N terminally truncated LSD1-CoREST. This change is visibly apparent upon comparing the fraction of demethylated H3K4me2 over time (Fig. 2, *E* and *F*). The disparity between the higher affinity SPR-derived dissociation constant for FL LSD1-CoREST and the enzymatic measurements on nucleosome substrates suggest that the FL LSD1-CoREST binds nucleosome substrates in an unproductive manner compared to ΔN LSD1-CoREST (19, 34).

### The NT-LSD1 region prefers binding to ΔN LSD1-CoREST and contains an **α**-helix

**Figure 3:**
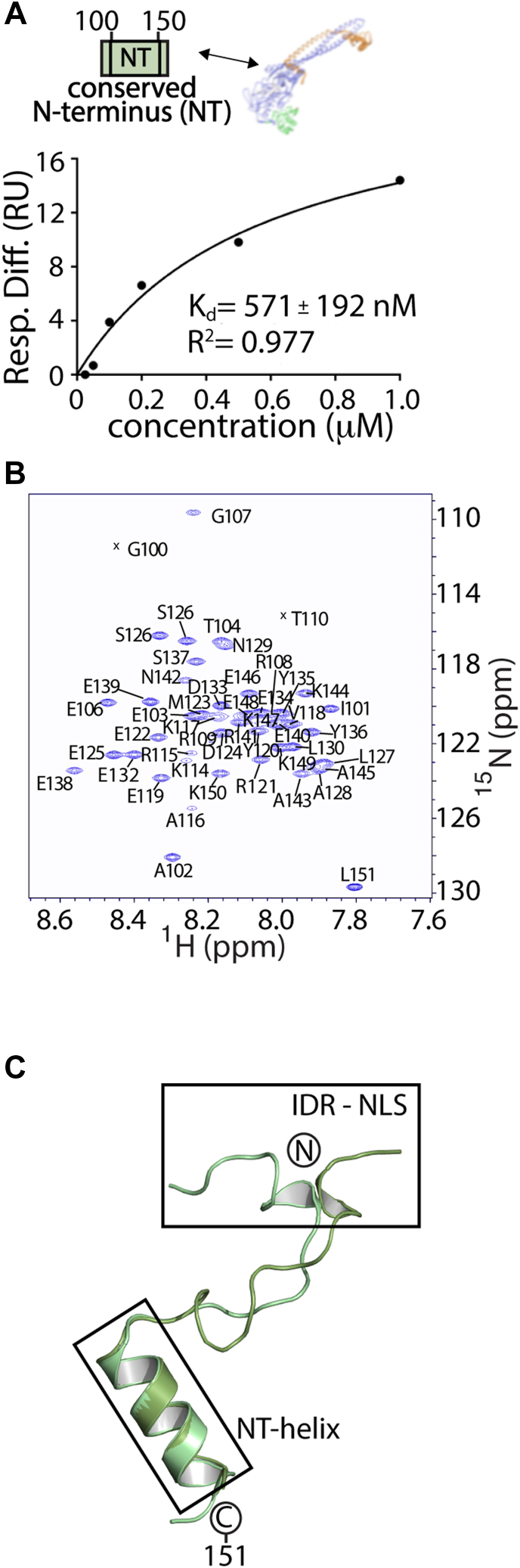
**Conserved N-terminal LSD1 region (NT-LSD1, 100–151) contains an α-helix and binds the ΔN LSD1 (171–852) – CoREST (286–482) complex.***A*, analysis of SPR data of a ΔN LSD1 complex to an immobilized NT-LSD1 polypeptide (aa. 100–151). The immobilized, biotinylated NT-LSD1 was subject to 25, 50, 100, 200, 500, and 1000 nM purified ΔN LSD1-CoREST. Analysis of SPR data revealed a binding dissociation constant *(K*_*d*_*)* for the conserved N terminus of LSD1 to ΔN LSD1. This NT-LSD1 polypeptide has much weaker, nonspecific binding to nucleosomes (Fig. S3). *Image disclosure*: Figure 3*A* is redundant with Fig. S5*B* and the same data were used to produce both figures. *Justification*: Figure 3*A* represents the analysis of raw sensorgram data in Support Fig. S5*A*. Reuse of Support Fig. S5*B* thereby consolidates all source data associated with this specific series of SPR experiments. *B*, ^15^N HSQC NMR spectrum (298 K) of a 0.5 mM ^15^N LSD1-NT polypeptide, with experimentally determined backbone amide resonance assignments (BMRB 27615). *C*, low energy predictions of the NT-LSD1 region (100–151) are shown with an identified helix (NT-helix, 135–148) forming within the conserved N-terminal region. Two representative models were generated from CS-Rosetta and subject to AMBER simulation (see structure calculation methods). Predicted structures corroborate experimental data from CD spectroscopy, ^15^N-NOESY-HSQC and Talos + NMR data (Fig. S6) and X-ray crystallography (PDB 6WC6). CoREST, corepressor for repressor element 1 silencing transcription factor; HSQC, heteronuclear single quantum coherence; LSD, lysine-specific demethylase; PDB, Protein Data Bank; SPR, surface plasmon resonance.

To further investigate binding interactions and transient structure within the N terminus, we overexpressed and purified the conserved N-terminal region (residues 100–151, termed NT-LSD1) to examine putative binding to the nucleosome, binding to ΔN LSD1-CoREST, and whether this NT-LSD1 (100–151) region contains any structural elements. The binding propensity between sequence of the NT-LSD1 (Table S1) and the nucleosome or to ΔN LSD1-CoREST was measured using SPR. Analysis of this experiment show that, while NT-LSD1 interacts weakly with nucleosomes in a nonspecific manner (Fig. S4), the NT-LSD1 (amino acids 100–151) binds to ΔN LSD1-CoREST with a dissociation constant (*K*_d_) of 571 ± 192 nM (Figs. 3*A* and S5). This suggests that the NT-LSD1 (a. a. 100–151) strongly prefers to bind ΔN LSD1-CoREST over nucleosomes.

In addition, CD spectroscopy of the NT-LSD1 (a. a. 100–151) reveals maximal negative ellipticity at 220 and 210 nm, indicative of global α-helical characteristics (Fig. S6*A*). To examine which residues are involved in α-helix secondary structure formation, NMR spectroscopy was performed on NT-LSD1. Analysis of triple-resonance experiments of a ^13^C-^15^N sample enabled the backbone assignments of 48 of 51 nonproline residues in the (^1^H, ^15^N) heteronuclear single quantum coherence (HSQC) spectrum (Fig. 3*B*, BMRB #27615). Most of the resonances were clear and well-defined in the spectra and TALOS-N analysis reveal the propensity for helix formation (Fig. S6*B*). In contrast, the 110 to 116 region of NT-LSD1 were too weak to be assigned, suggesting μs-ms timescale dynamics. To determine the precise secondary structure, ^15^N-NOESY-HSQC spectra were acquired and proton-amide proton NOEs were identified (Fig. S6*C*). A series of short-range and medium-range interresidue NOE resonances were unambiguously assigned spanning Y135 to E146, including assignments of HN-HN (i, i + 1), HN-HN (i, i + 2), HN-HN (i, i + 3), H_α_-HN (i, i + 2), and H_α_-HN (i, i + 3). These assignment patterns identify the formation of a stable α-helix, spanning residues 135 to 148. Other NOEs, such as HN Y135-HN E139, H_α_ S131-HN Y136, and H_α_-HN (i, i + 1) connectivities, provide additional evidence for a stable helix within this region. These NMR results show high consistency with secondary structure prediction determined by Talos-N, which incorporated backbone (N, HN, C, C_α_, H_α_, and C_β_) derived chemical shifts (Fig. S6*B*) (35).

To further verify this structural element, a Rosetta *de novo* prediction and MD simulation were applied to NT-LSD1 (a. a. 100–151). Chemical shift data from ^15^N-HSQC, HNCACB (36), and CBCA(CO)NH (37) experiments were used to guide structure prediction with CS-Rosetta, and structures generated with CS-Rosetta revealed an α-helix spanning residues 135 to 148 that remains stable over 20 independent 10 μs MD simulations (Fig. S6*D*, Tables S2 and S3). The residues form a basic patch and a negatively charged surface on either face of the α-helix. Although residues across 100 to 151 failed to converge in the CS-Rosetta prediction, *ab initio* structures showed convergence and ten independent 2 μs MD simulations from the top-scoring models showed that the region spanning residues 135 to 148 contains α-helical character. A total of six independent 2 μs MD simulations across the best scoring models from *ab initio* Rosetta clusters demonstrate the stability of an α-helix (Table S3, Movie SM1). This Amber-based molecular modeling simulation shows that the LSD1 region 100 to 117 adopts transient helical character but is not stable, whereas the 135 to 148 region retains a more stable α-helix throughout the course of the simulation (Fig. 3*C*).

### The NLS of LSD1 inhibits H3K4me2 demethylation on peptide substrates

To determine the exact regions of N terminal sequence that bind or may influence LSD1 catalyzed demethylation, short peptides across the highly conserved region (a.a. 103–151) of LSD1 N terminus were prepared. Peptides include the NLS peptide (positively charged, a.a. 107–120, pI ∼11.84, termed “NLS”) that contains the NLS sequence, a negatively charged peptide region (a.a. 121–136, pI ∼3.77, termed “P1”) and a neutral-charged α-helical containing peptide region (a.a. 137–151, pI ∼6.07, termed “P2”) (Table S1).

**Figure 4:**
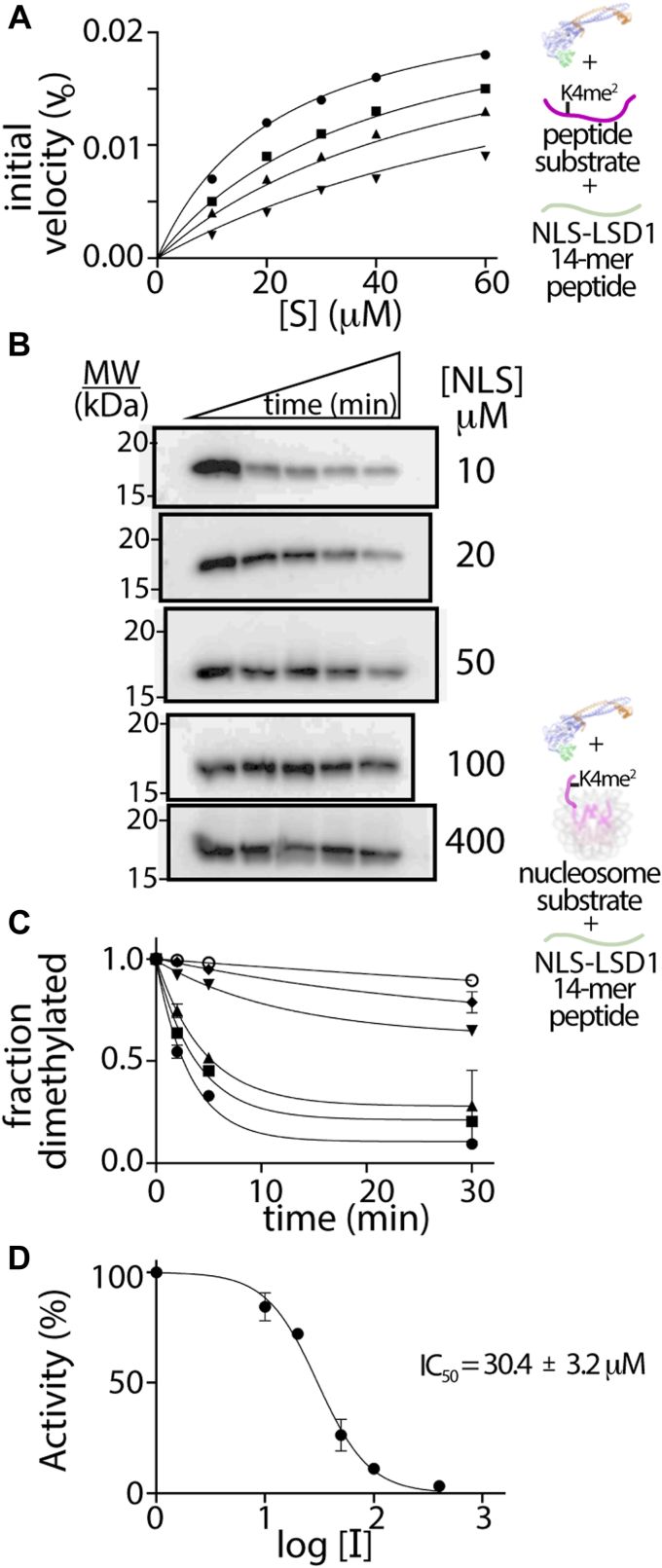
**Autoinhibition of demethylation by LSD1’s own nuclear localization signal region.** ΔN LSD1 demethylation on an H3K4me2 peptide substrate in the presence of three peptides spanning the conserved N terminus of LSD1 was performed (Fig. S7), yet only A) the NLS containing peptide (aa 107–120, NLS-LSD1 (*green*)) resulted in enzyme inhibition (K_i_^app^ ∼ 3.3 ± 0.6 μM, Table S2) on model peptide substrates. A K_i_^app^ value was determined based upon NLS-LSD1 peptide concentrations (0 μM (*circles*), 2.5 μM (*squares*), 5 μM (*triangles*), and 10 μM (*inverted triangles*)) and various H3K4me2 peptide substrate concentrations (10, 20, 30, 40, and 60 μM) (N = 2). *B*, Western blot assays showing ΔN LSD1 catalyzed demethylation on H3K4me2 nucleosome substrates as probed using an anti-H3K4me2 antibody in the presence of increasing NLS-LSD1 peptide (10–400 μM) over time (0, 2, 5, 30, and 120 min). *C*, quantification of blot image intensities based upon the relative fraction of dimethylated nucleosomes from anti-H3K4me2 and control anti-H3 antibodies. The *k*_*observed*_ parameters were determined for 0 (*black circle*), 10 μM (*square*), 20 μM (*triangle*), 50 μM (*inverted triangles*), 100 μM (*diamonds*), and 400 μM (*open circles*) NLS-LSD1 peptide concentrations (N = 2). *D*, relative percent activity (%) *versus* inhibitor concentrations (log [I]), revealing the IC_50_ (30.4 ± 3.2 μM) for the NLS-LSD1 peptide on LSD1-based demethylation on H3K4me2 nucleosomal substrates. H3K4me2, histone 3 lysine 4 dimethyl; LSD, lysine-specific demethylase; NLS, nuclear localization signal.

An inhibition study was performed using horseradish peroxidase (HRP) coupled demethylase assay in the presence of NLS, P1, or P2 peptides, using the 21 a.a H3K4me (2) peptide as substrate (9, 38). Of the three peptides, only NLS peptide (a.a. 107–120) acted as an inhibitor of ΔN LSD1-CoREST (Fig. 4*A*), whereas the P1 and P2 peptides did not influence the demethylation reaction (Fig. S7). When a full inhibition study was performed using different concentrations of the 21 a.a. H3K4me (2) peptide as substrate and different concentrations of NLS peptide inhibitor, an inhibition constant (*K*_*i*_) of 3.3 ± 0.6 μM was determined (Fig. 4*A*, Table S4). This suggests that the NLS peptide is an inhibitor of LSD1 catalytic activity with comparable inhibition to other known peptide fragments whose proteins are known to directly interact with LSD1 (Table S4). Control experiments indicate that the NLS peptide directly influences the LSD1 catalyzed reaction and that inhibition is not an artifact of the HRP-coupled assay system (Fig. S8). In addition, analysis of the Lineweaver Burk plot suggests that the NLS peptide serves as a competitive inhibitor of demethylation, indicating direct binding near the ΔN LSD1-CoREST active site (Fig. S9).

### The NLS of LSD1 inhibits H3K4me2 demethylation on nucleosome substrates

Based on these coupled assay results and data (Fig. 2, *C* and *D*), we developed an *in vitro* quantitative WB assay to monitor LSD1-catalyzed demethylation on H3K4me2 nucleosome substrates. This approach provides enhanced reliability of the antibody reagents as well as a robust measure of the impact of the NLS peptide on LSD1’s ability to demethylate nucleosomes (39). A series of time-course assays with increasing NLS peptide measured the fraction of demethylated H3K4me2 nucleosomes containing a 147 bp DNA, and this was visualized using an anti-H3K4me2-specific antibody or a control H3 antibody (Fig. 4, *B* and *C*). Here, dividing the H3K4me2 antibody signal by the H3 antibody signal allowed for quantitation of each data point, where the disappearance of H3K4me2 signal directly corresponds to the NLS's ability to impact LSD1’s demethylation activity. The H3K4me2/H3 ratios were normalized at time zero and plotted as a function of time (minutes), and the determined rate constant (k_obs_) values were evaluated at increasing NLS concentrations (Fig. 4, *B* and *C*). The IC_50_ parameters for NLS inhibition of LSD1 activity were determined (IC_50_ = 30.4 ± 3.2 μM) based on the following equation:

$$\text{Activity}\;\left(\%\right)=100/\left(1+10^{\left(\mathrm{Log}\;\text{IC}50-\left[I\right]/n\right)}\right)$$

where [I] is the log of dose (log [I]), the relative determined rate constant (k_obs_) with and without inhibitor is defined as activity (%), and the Hill coefficient (*n*) is estimated as one, corresponding to 1:1 LSD1:NLS binding (Fig. 4*D*). One reason for the different inhibitory effects of the NLS on short peptide [*K*_*i*_ of 3.3 ± 0.6 μM] *versus* nucleosome substrates IC_50_ = 30.4 ± 3.2 μM is because the nucleosome, which contains both anionic (DNA) and electropositive (histone) features, likely binds and neutralizes the electropositive NLS peptide. Nonetheless, these data support a role for electrostatic-based inhibition of LSD1 demethylation by its own NLS.

### Phosphomimic NLS mutations alleviate inhibition of LSD1-based demethylation

To better understand how the electropositive NLS region influences LSD1 catalyzed demethylation, relevant PTMs or phosphomimetic substitutions were incorporated into the NLS peptide and tested using model and nucleosome substrate activity assays. Just as the H3 N-terminus contains an abundance of PTMs(Fig. 1*C*), the highly conserved NLS region also contains functional phosphorylation (T110/S111) and dimethylation (K114) sites that span the NLS region. Both PTM-mimetic NLS peptides and a full length LSD1 containing phosphomimetic (T110D/S111D) mutations serve as strategies to examine how PTMs near the NLS region impact LSD1 catalyzed demethylation.

For the demethylase-HRP coupled peroxidase assays, inhibition of demethylation activity was retained after inclusion of a 10 μM dimethylated K114 peptide construct (aa 107–120, K114me2, termed NLS-K114me2). In addition, the NLS-K114me2 peptide does not serve as a substrate, as previously demonstrated (23). However, the presence of 10 μM of a phosphomimic NLS peptide (aa 107–120 (T110D/S111D, NLS-p∗) do not obstruct LSD1-catalyzed demethylation (Fig. S10*A*). This NLS-p∗ result contrasts with the inhibitory effect of the NLS peptide (Figs 4*A* and S7). A complete profile of the NLS-p∗ peptide suggest that introducing an electronegative phosphate at the specific location adjacent to the NLS sequence partially relieves inhibition on a 21aa H3K4me2 model peptide substrate (K_i_ = 38.7 ± 1.8 μM), approximately 12× weaker compared to the NLS peptide (Fig. 4*A*, Table S4). These data confirm that the presence of different PTMs near NLS region can differentially influence the LSD1 demethylation reaction.

**Figure 5:**
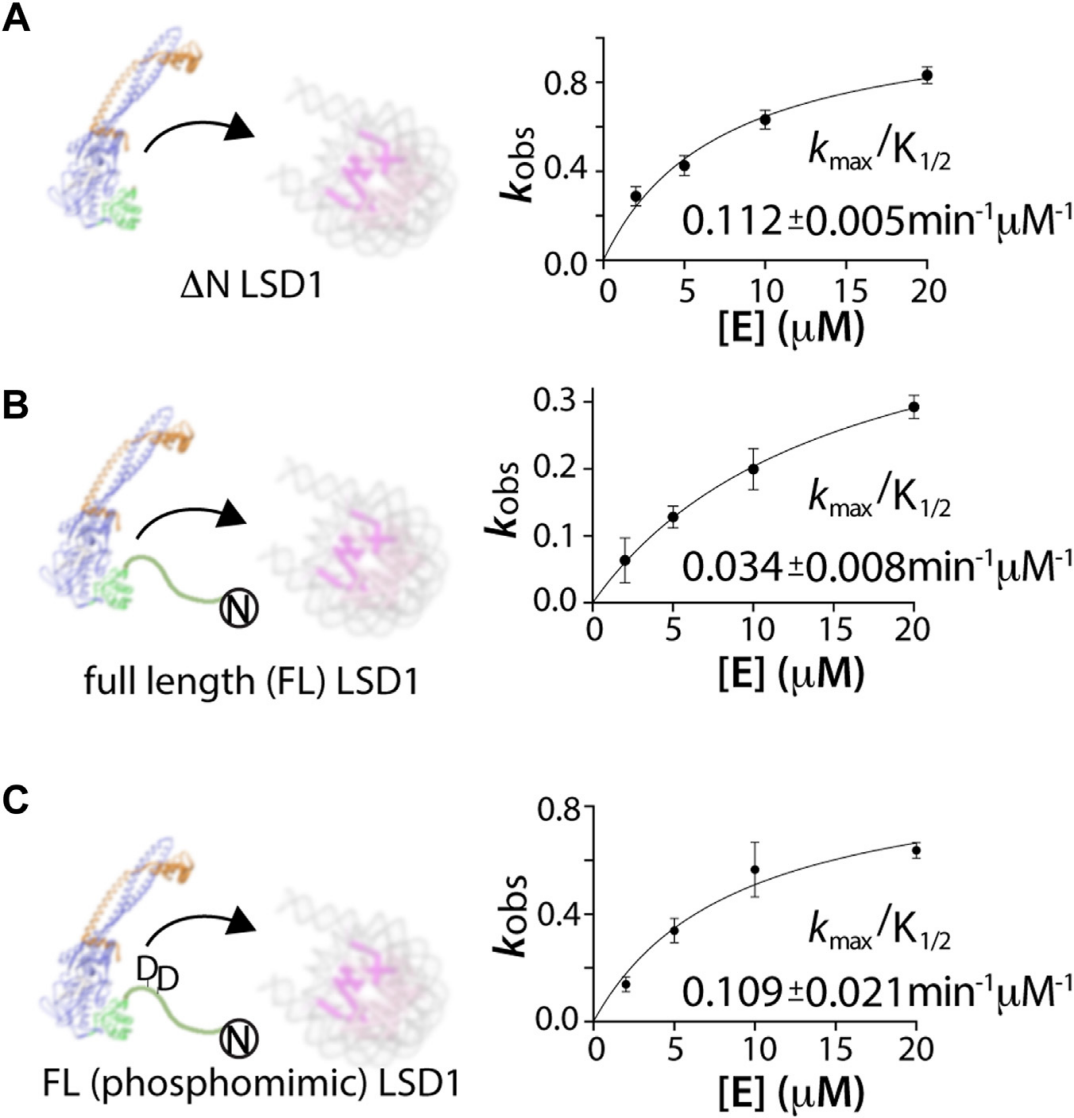
**Comparison of the catalytic efficiency of LSD1 constructs on H3K4me2 nucleosomal substrates suggests phosphorylation of T110/S111 partially relieves autoinhibition.** A plot of *k*_*observed*_*versus* enzyme [E] concentration enables the determination of comparative *k*_*max*_*/*K_1/2_ values for (*A*) ΔN LSD1, (*B*) full-length (aa 1–852) LSD1, and (*C*) an T110D/S111D full-length (aa 1–852) LSD1 on H3K4me2 nucleosome substrates. (Fig. S11). For *A*–*C*, A schematic of the ΔN LSD1-CoREST, full-length LSD1-CoREST, and full-length LSD1 (T110D/S111D)-CoREST with LSD1 (*blue*/*green*) and CoREST (*orange*) engaging with nucleosomes (DNA (*gray*) and histones (*pink*, H3 and *light pink* (all other histones)) is shown. CoREST, corepressor for repressor element 1 silencing transcription factor; H3K4me2, histone 3 lysine 4 dimethyl; LSD, lysine-specific demethylase.

To validate whether this phosphomimetic substitution influences the activity in the context of FL LSD1-CoREST, site directed mutagenesis was performed on the FL LSD1 construct, introducing T110D and S111D mutations proximal to the NLS sequence. The kinetics of demethylation reaction of mutant FL LSD1-CoREST (T110D/S111D) on nucleosome substrates was measured using single turnover conditions measured by quantitative WBs using anti-H3K4me2 and H3 antibodies. The time course of LSD1 catalyzed demethylation was used to measure dimethylated H3K4me2 at each time point, and data from blots were normalized using corresponding H3 antibody signal monitored by anti H3 antibody. Interestingly, the determined catalytic efficiency (*k*_*max*_/*K*_*1/2*_) values for mutant FL LSD1-CoREST (T110D/S111D) (*k*_*max*_/*K*_*1/2*_ = 0.109 ± 0.021 min^−1^ μM^−1^) were comparable to *k*_*max*_/*K*_*1/2*_ values of the ΔN LSD1-CoREST on nucleosomes (0.112 ± 0.021 min^−1^ μM^−1^) and are three-fold higher than the FL LSD1-CoREST (0.034 ± 0.008 min^−1^ μM^−1^) (Figs 5 and S11). The comparison of the catalytic efficiencies of different LSD1 constructs on H3K4me2 nucleosomes provides additional evidence that phosphorylation of T110 or S111 relieves the autoinhibitory properties of the NLS region.

### Comparative 20 μs MD simulation of H3 and NLS peptides at LSD1’s active site

Due to the competitive inhibitory properties of the NLS peptide in LSD1 demethylation, the sequence similarities between the H3 tail (pI 12.3) and LSD1’s NLS regions (pI 11.8), and the functionally relevant PTMs shared between the NLS and the human H3 tail (Fig. 1, *B* and *C*), we surmised that the NLS region interacts near LSD1’s large active site cleft. Thus, we pursued a combined X-ray crystallography and MD simulation approach to identify the binding location of the NLS-LSD1 interface.

Crystals of ΔN LSD1-CoREST were soaked in the presence of the NLS peptide (2–10 mM) and this yielded low resolution 3.5 Å diffraction data sets. Cocrystallization trials were also pursued but unsuccessful. Analysis of the difference density (mFc-DFc map) across the LSD1-CoREST structure identified the binding location of the NLS peptide on the surface of LSD1 (Fig. S12). The weak difference density at the active site cleft was attributed to the NLS peptide because this was the only difference between the composition of the crystallization and crystal soaking conditions. Thus, an atomic level depiction of the LSD1-NLS interface could not be unambiguously resolved due to the poor resolution of the data, despite exhaustive crystallographic trials and variations of solution conditions. The accurate placement and orientation of the NLS peptide was also not possible given the likely transient difference density of the largely electrostatic-based interaction, owning to the crystal soaking conditions performed under high anionic (1.2–2 M formate) crystallization conditions. Nonetheless, a model of the NLS peptide was manually docked into the difference density in two different orientations based upon this difference density and the LSD1-H3 tail nucleosome structure (Protein Data Bank (PDB) 6VYP). These crystallography data suggest that the NLS peptide can localize at LSD1’s active site cleft.

**Figure 6:**
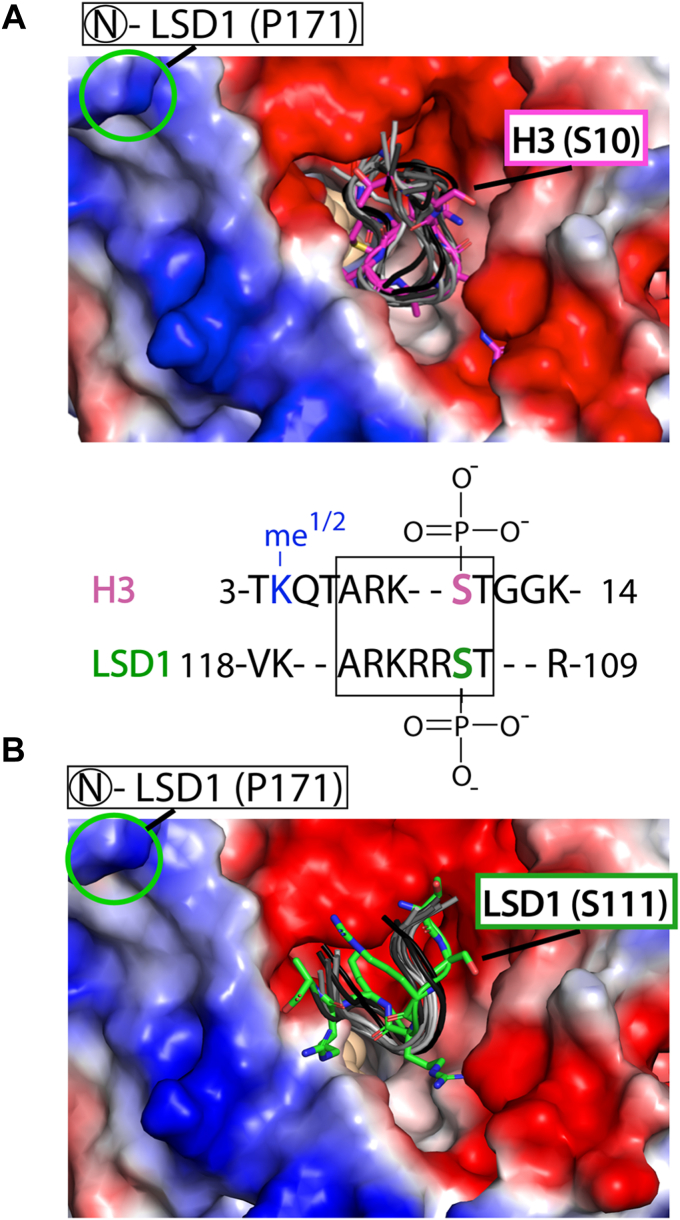
**Comparative 20 μs molecular dynamics simulation of histone 3 and NLS peptides near the LSD1 active site pocket.***A*, APBS electrostatic surface map of the LSD1 structure (*red*, electronegative, to *blue*, electropositive, with ramp levels −64.132–64.132 in complex with nucleosome H3 tail (*pink*). Structure from PDB 6VYP. Ten trajectory models (*gray-black backbone cartoon*) of the H3 tail (aa 3–11) spanning the 20 μs Amber simulation inform on the conformational dynamics within the active site pocket. A comparison of the primary sequence and N to C terminal directionality of the H3 (aa 3–14) with the reverse directionality of the human LSD1’s N-terminal region (aa 109–118) are shown. The locations of methylation (*blue*) and the phosphorylations on H3 and LSD1 are noted and where the approximate phosphorylation sites could be positioned is highlighted in the (*A*) and (*B*). *B*, APBS electrostatic surface map with ten trajectory models (*gray-black backbone cartoon*) spanning an identical 20 μs Amber simulation with the NLS peptide (LSD1, aa 110–117, *green*). Initial placement of the NLS peptide at the surface of LSD1 was derived from crystal soaking studies, where high salt LSD1-CoREST crystals were soaked with an NLS peptide. This experiment revealed weak difference density, indicative of low occupancy NLS ligand binding (Fig. S12*A*). It is likely that high salt crystal conditions contribute to the low occupancy of the electrostatic-based NLS-active site interaction. Nonetheless, a 20 μs Amber simulation informed on the “residence” time and relative stabilization of the NLS peptide near LSD1’s active site pocket. CoREST, corepressor for repressor element 1 silencing transcription factor; LSD, lysine-specific demethylase; NLS, nuclear localization signal; PDB, Protein Data Bank.

Subsequently, models of the docked NLS-LSD1 orientations and the known H3 tail-LSD1 structure (6VYP) were subject to 20 μs AMBER based MD simulations, allowing for a direct comparison of the trajectories and a measure of peptide ligand stability within the enzyme active site cleft. For the H3 tail (aa 3–11), ten trajectory models spanning a 20 μs AMBER simulation informed upon the conformational flexibility of the substrate bound within the active site and yield a backbone RMSD of 1.65 ± 0.03 Å (Fig. 6*A*). This result is comparable to a GROMACS-based MD simulations assessing the conformational diversity of a 16 aa H3 tail bound to the LSD1 active site (RMSD 1.9 Å) (40). In addition, identical AMBER MD simulations of the NLS tail (aa 110–117) docked at the active site revealed that one orientation is conformationally preferred and that the ten trajectory models are clustered together (backbone RMSD of 1.81 ± 0.13) near the active site pocket of LSD1 (Fig. 6*B*). Taken together, this comparative MD simulation provided a structural model for how LSD1’s own NLS can interact near the active site cleft, contributing to electrostatic-based inhibition.

## Discussion

The dynamic N-terminal tail of LSD1 contains electropositive and electronegative clusters recognized as unstructured elements, yet this region serves as a key protein-protein interaction hub with extensive PTMs and an NLS region that regulates import into the nucleus and transcription regulation (23, 24, 26, 28, 41, 42, 43). Interestingly, LSD1-catalyzed demethylation functions primarily on the histone tails of nucleosomes, which themselves consist of electropositive IDRs that undergo extensive PTMs that enable the recruitment or exclusion of proteins to influence the packing of chromatin. Our data reveal that the electropositive NLS region within the conserved 45 amino acid IDR element of LSD1 serves as a tunable element to bind near the amine oxidase active site and influence LSD1’s catalytic efficiency on H3K4me2 peptide and nucleosome substrates. Additionally, incorporation of phosphomimetic mutations within the NLS region substantially alleviate this inhibition, suggesting a regulatory role for phosphorylation adjacent to LSD1’s NLS. Taken together, we propose that the NLS region of LSD1 can mimic the H3 tail in an electrostatic-based manner to fine-tune and mediate LSD1-catalyzed demethylation on nucleosomes, thereby acting as an autoregulatory element.

A precedence for this autoregulatory model in LSD1 is supported through recent biochemical and cell-based studies that examine LSD1’s protein-protein interaction hub (28). Specifically, the N terminus of LSD1 (a.a. 1–170) was found by Waterbury et al., (28) to engage with its adjacent structured domains through a series of transient interactions, thereby establishing a cell-based molecular function for this IDR element to act as a “fuzzy” molecular switch, regulating LSD1-transcription factor (GLF1B, C/EBPa, RUNX1, and PU.1) interactions, modulating silencing-enhancer activity across cis-regulatory landscapes, and impacting AML cancer-associated differentiation. In addition, there is precedence for a phospho-mediated autoregulatory model in LSD1 that is supported through previous biochemical studies. For example, phosphorylation of the serine 10 site of H3 (H3S10p) is a mark associated with the initiation of cancer and was shown to strongly impede LSD1 demethylation on model substrates by an order of magnitude (Table S4) (9). This seminal work showed how phosphorylation and IDRs influence H3K4me2 demethylation and emphasized the importance of electrostatic interactions in LSD1-substrate recognition. Similarly, our data reveal that phosphomimetic substitutions impede NLS-mediated inhibition by over 10× fold on model substrates and nearly restore full-length enzyme activity on nucleosome substrates. This unanticipated connection suggests a biophysical mechanism whereby the NLS transiently interacts to impede demethylation activity and that PTM-mediated NLS-phosphorylation attenuates the role of the N-terminal IDR during catalytic activation.

Previous studies have also demonstrated the importance of PTMs near the NLS region, with key functional roles that includes the following: enhancing binding to HDAC1, regulating stability of p65 in the activation of the inflammation response, and inducing circadian genes (CLOCK and BMAIL1) to facilitate E-box transcriptional activation (26). Specifically, phosphorylation of S111 influences the promotion of the epithelial-mesenchymal transition (EMT), and this specific PTM is known to decrease the repressive activity of LSD1, which is associated with breast cancer stem cell development. In addition, immunotherapy-resistant melanoma patients also express higher levels of phosphorylated S111 LSD1, similarly modulating stem-like EMT signatures and altering LSD1’s transcription regulation network in cancer-specific cells (42, 44).

Although this region and the roles of N-terminal PTMs have primarily been suggested to influence LSD1’s protein-protein network, we demonstrate evidence that the electropositive NLS (aa 107–120) can fine-tune LSD1 activity and can autoregulate histone demethylation on nucleosome substrates. Additionally, we identified a novel α-helical N-terminal element (aa 138–148) using multiple biophysical approaches, yet this transient helix does not appear to impact the demethylation and its functional role awaits further investigation. Taken together, comparative kinetic and MD simulations suggest a molecular mechanism for how the NLS (aa 107–120) acts as a competitive inhibitor in the demethylation reaction and how NLS-based phosphorylation can potentially regulate electrostatic-based autoinhibition. This autoregulatory model will assist in developing stronger dual inhibitors that target enzyme activity and the selective phosphorylation pathways of LSD1’s N terminus that target stem cell-like EMT signatures. More broadly, this work introduces the novel concept that phosphorylated NLS regions may not only regulate nuclear transport (45, 46, 47), but may also fine-tune chromatin modifying activity in an autoinhibitory manner.

## Experimental procedures

### Overexpression and copurification of human LSD1-CoREST

The N terminally truncated (aa 171–852) LSD1 plasmid (pGEX-6P-1-ΔN LSD1) and pET28-CoREST plasmid (aa 286–482) were generous gifts of Dr Cole (Johns Hopkins University) and full-length (FL) LSD1 (aa 1–852) plasmid (pET-15b-FL LSD1) was a generous gift of Dr Shi (Harvard University). Full-length or truncated LSD1 plasmid and CoREST plasmid were coexpressed in Rosetta (DE3) pLysS competent cells. For overexpression of LSD1-CoREST, cells were grown at 30 °C in auto induction media containing 200 μg/ml ampicillin, kanamycin, and 40 μg/ml chloramphenicol antibiotic concentrations. The expressed ΔN LSD1-CoREST was purified by Ni-affinity chromatography and GST affinity chromatography before 3C protease and thrombin digestion followed by Ni-affinity chromatography, GST affinity chromatography, and Superdex 200 size-exclusion chromatography. The expressed FL LSD1- CoREST was purified by Ni-affinity chromatography before thrombin digestion followed by Superdex 200 size-exclusion chromatography. Protein was stored in 25 mM Hepes Na pH 7.5, 100 mM NaCl, and 5 mM Tris(2-carboxyethyl)phosphine (TCEP) buffer.

For the N terminal phosphomimetic, termed FL LSD1 (FL DD LSD1) and containing the T110D S111D point mutations, site-directed mutagenesis was performed on the pET-15b bacterial expression vector carrying FL LSD1 (1–852). The plasmid was PCR amplified and the CloneAmp HiFi kit was used to incorporate the forward and reverse primers (5′ – aggggcgt cgggacgaccggcgcaagc-3′ and 5′- gcttgcgccggtcgtcccgacgcccct -3′, respectively). Upon Dpnl restriction digestion, the DNA products were subject to a GeneJET DNA cleanup kit (Thermo Fisher Scientific) and the purity of the product was visualized by 0.8% agarose gel electrophoresis. After transformation in DH5α competent cells, cell propagation on LB-agar plates and LB media (100 mg/ml ampicillin), and a GenElute HP plasmid miniprep kit, the mutations in the pET-15b plasmid were confirmed using DNA sequencing. The FL LSD1 (FL DD LSD1) protein was subsequently overexpressed and purified as outlined above.

For all purified LSD1-CoREST constructs (ΔN LSD1, FL LSD1, and FL DD LSD1), the concentration of protein samples was determined by the bicinchoninic acid method (BCA Protein Assay kit, Pierce) with bovine serum albumin as a standard and by UV-VIS-spectroscopy for LSD1 preparation with extinction coefficient for FAD at 450 nm as 11,300 M^−1^ cm^−1^. Protein spectra were recorded on Shimadzu UV-2600 Spectrophotometer and protein purity was assessed by SDS-PAGE with Coomassie staining.

### LSD1 N terminus cloning, expression, and peptide purification

The N-terminal fragment of LSD1 (NT-LSD1, residues 100–151) with sequence: GIAETPEGRRTSRRKRAKVEYREMDESLANLSEDEYYSEEERNAKAEKEKKL was cloned into pBG101 expression vector (Vanderbilt University). The fragment was PCR amplified with forward primer 5′CTAGTAGGATCCGGAATAGCAGAGACTC-3′, which contain BamHI restriction site and reverse primer 5′-CGTAGTGCTAGCTCAAAGCTTCTTTTCCTTC-3′ which contains NhaI restriction site. The prepared PCR product was inserted into BamHi-NheI sites of pBG101 vector. LSD1-NT fragments were expressed as fusion proteins with N-terminal GST and His-tags.

NT-LSD1 was expressed in *Escherichia coli* BL21 (GOLD) cells in LB growth media with kanamycin and 1 mM IPTG at 37 °C for 5 to 6 h. Harvested cells were homogenized by ultrasonic treatment in 50 mM Tris, pH 7.5 to 150 mM NaCl buffer with 2 mM PMSF, 1 mg/ml leupeptine and 1 mg/ml pepstatin as protease inhibitors. An EDTA-free tablet (Roche cOmplete) was alternatively applied as a protease inhibitor cocktail. Homogenate was subsequently centrifuged (18,000 rpm, 40 min), and the supernatant was applied to a Ni-NTA column for NT-LSD1. After washing with 8 to 10 column volumes of 50 mM Tris, pH 7.5 and 150 mM NaCl buffer, NT-LSD1 was eluted with 250 mM imidazole. The NT-LSD1 fraction was dialyzed in 50 mM Tris, pH 7.6, 150 mM NaCl, and 1 mM DTT concentrated, and subsequently purified using size-exclusion chromatography. The NT-LSD1 proteins were treated with PreScission Protease overnight at 4 °C, enabling the cleavage of the His-GST tags. Separation of the LSD1-NT and the His-GST tag was achieved using affinity chromatography (Ni-NTA or GSH 4B). The fractions in the wash contained pure NT-LSD1and were pooled and concentrated in Amicon Ultra 3K Centrifugal Filters (Millipore).

In all NMR studies, a ^13^C-^15^N uniformly labeled NT-LSD1 was prepared using the identical purification procedure, with the following changes. Overexpression of NT-LSD1 in *E. coli* BL21(GOLD) cells was performed in M9 minimal media (^15^NH_4_Cl and ^13^C-glucose) containing 200 μg/ml kanamycin. The NMR protein samples were dialyzed again 2 L of NMR buffer (5 mM Tris (pH 7.6), 150 mM NaCl) and subsequently concentrated to 0.3 to 0.8 mM using an Amicon Ultracellulose Centrifugal Filter (3K, MWCO) (Millipore).

Short, peptide fragments within the NT-LSD1 region were purchased from GenScript and contained contain acetylation and amidation modifications at the N and C termini, respectively. The peptide fragments (>95% purity) examined in biochemical assays included the following: GRRTSRRKRAKVEY (NLS-NT, 107–120), REMDESLANLSEDEYY (P1 peptide, 121–136), YSEEERNAKAEKEKK (p2 peptide, 136–151), GRRDDRRKRAKVEY (NLSp∗, 107–120), and GRRTSRRKme2RAKVEY (NLS-K114me2, 107–120). All peptides were dissolved in the identical buffer (25 mM Hepes pH 7.5, 5% glycerol, 1 mM TCEP, and 50 mM KCl), desalted and were treated in an identical manner in activity assays.

### SPR binding assays

To examine LSD1-CoREST binding to the nucleosome, biotinylated recombinant human nucleosomes (EpiCypher) were immobilized at surface densities between 4000 and 5000 RU. ΔN LSD1-CoREST was titrated with the following concentrations: 5, 10, 25, 50, 100, 200, 300, 400, 500, and 2000 nM concentrations. Full-length LSD1 (residues 1–852) with CoREST (286–482) was injected at the identical concentration points as ΔN LSD1-CoREST until 500 nM, as aggregation occurs at higher concentrations. ΔN or FL LSD1-CoREST were injected at a flow rate of 40 μl/min for 2 min, at various concentrations (above). The time course of dissociation was recorded for 4 min. All SPR measurements were performed on a Biacore 3000 instrument (GE HealthCare) at 298K in running buffer (10 mM Tris, 100 mM NaCl, 1 mM EDTA, 2 mM TCEP, and 0.005% Tween 20, pH 7.4). At the end of each measurement, a regeneration of mononucleosome surfaces was conducted by applying a mixture of 0.01% Tween 20 and 0.1% NP40 diluted in running buffer (4 consecutive injections for 30 s), and subsequently equilibrated with running buffer (5 min). For each concentration for each LSD1 variant, at least two measurements were collected.

To examine the NT-LSD1 binding to nucleosomes, a biotinylated nucleosomes was immobilized as described above and was subjected to 0.1, 0.5, 1, 2, 3, 5, 8, and 10 μM NT-LSD1 concentrations (N = 2). An analysis of RU *versus* NT-LSD1 ligand concentrations suggest a weak binding interaction (Fig. S4) in running buffer conditions (10 mM Tris, 100 mM NaCl, 1 mM EDTA, 2 mM TCEP, and 0.005% Tween 20, pH 7.4). For all nucleosome binding SPR studies, biotinylated nucleic acid (Widom 601) was immobilized on a streptavidin-coated sensor chip SA (GE HealthCare). Streptavidin surfaces saturated only by biotin served as a reference for all collected data.

For characterizing binding of NT-LSD1 to LSD1-COREST, biotinylated NT-LSD1 was immobilized on the sensor chip with a surface density of 560 RU. Biotinylation of the NT-LSD1 peptide was generated by the EZ-Link Sulfo-NHS-LC-Biotinylation Kit (Thermo Fisher Scientific) and purified *via* SpinOUT GT-100 1 ml column (G-Biosciences). ΔN LSD1-CoREST was injected at a flow rate of 40 μl/min for 2 min in running buffer conditions, with various concentrations of 25, 50, 100, 200, 500, and 1000 nM. The time course of dissociation was recorded for 4 min. At the end of each measurement, regeneration of the NT_LSD1 surface was conducted by applying a mixture of 0.01% Tween 20 and 0.1% NP40 diluted in running buffer (4 consecutive injections for 30 s), and subsequently equilibrated with running buffer (5 min).

Data processing and evaluation were performed in BIAevaluation 4.1.1 (Biacore AB, https://www.cytivalifesciences.com/). The kinetic curves were fitted using the 1:1 Langmuir binding mode to determine k_a_, k_d_, and K_d_ (Fig. S2). For the titrations which were binding-saturated during the association periods, analysis of the binding specificities were also determined by plotting the RUs at the saturated *versus* protein concentrations in Prism 6.01 (GraphPad Software) (http://www.graphpad.com), and analyzed using the equation:

[1] $$\text{RU}={\text{RU}}_{\max}*C\wedge h\;/\;\left(K_{d}\wedge h+C\wedge h\right)$$

Where RU_max_ is the maximum binding response units, C is the ligand concentration, h is the Hill slope and K_D_ is the equilibrium dissociation constant when h = 1. While the SPR experiment reveals that the N terminus is necessary for full binding to nucleosomes, it is possible that the N terminus may contribute to enhanced binding through an indirect (allosteric or nonspecific ionic) binding mechanism. It is also likely that nonconserved LSD1 residues 1 to 99 contribute to binding mononucleosomes.

### Demethylation activity using model substrates in the presence of N-terminal peptide fragments

LSD1-CoREST activity assays were performed using well-established peroxidase-coupled assay that monitors H_2_O_2_ production under aerobic conditions (48). Initial velocity measurements were measured using a Shimadzu UV2600 UV-Visible spectrophotometer equipped with thermostated cell holder (T = 25 ˚C). ΔN LSD1-CoREST was buffer exchanged into 25 mM Hepes pH 7.4, 100 mM NaCl, and 5 mM TCEP buffer. Each 150 μl reaction mixture contained 50 mM Hepes (Na) pH 7.5, 0.3 μM ΔN LSD1-CoREST, 1 μg of HRP, 0.1 mM 4-aminoantipyrine, and contained various substrate concentrations (10, 20, 30, 40, and 60 μM). Enzyme reactions were initiated by the addition of substrate; H3K4me2 peptide aa 1 to 21 or the NLS-K114me2 peptide (107–120, dimethylated at K114), into the reaction mixture in quartz cuvette. Activity region monitored was 0 to 30 s, and total time of each measurement was 180 s. Baseline correction was done at 505 to 525 nm. Changes in absorbance were measured at 515 nm wavelength and initial velocity calculations were done using an extinction coefficient of 26,000 M^−1^ cm^−1^ by Graphpad Prism (http://www.graphpad.com). Initial velocity values obtained from absorbance *versus* time graph, and they were fitted to Michaelis-Menten equation nonlinear regression and obtained V_max_ (maximum velocity), apparent *k*_*max*_ (turnover number) and K_m_ (Michaelis constant).

Peptide inhibition assays in presence of model substrate were conducted as follows using a peroxidase-coupled assay that monitors H_2_O_2_ production under aerobic conditions. A 150 μl reaction mixture contained 50 mM Hepes (Na) pH 7.5, 0.3 μM ΔN LSD1-CoREST, 1 μg of HRP, 0.1 mM 4-aminoantipyrine, 1.0 mM 3,5-dichloro-2- hydroxybenzenesulfonic acid, NLSK114me2 10 μM (as inhibitor and substrate)/NLSp∗ 10 μM/NLS 10 μM/P1 10 μM/P2 10 μM and H3K4me2 21aa substrate 10 μM. Enzyme reactions were initiated by the addition of substrate; H3K4me2 peptide aa (1–21) into the reaction mixture in quartz cuvette. Initial velocity values obtained from absorbance *versus* time graph were fitted to inhibition models using nonlinear regression fit. Complete kinetics profiles were performed for the NLS and NLSp∗ peptides, where different concentrations of NLS (0, 2.5 μM, 5 μM, 10 μM) or NLSp∗ (0, 10 μM, 40 μM, 100 μM) were incubated with freshly prepared ΔN LSD1-CoREST. Data from both the NLS and NLSp∗ activity assays on model substrates were fit to competitive inhibition profiles, enabling determination of Ki (inhibitor constant) values.

### Demethylation activity using H3K4me2 nucleosome substrates

Quantitative WB activity assays of the ΔN LSD1- CoREST and FL-LSD1- CoREST at 2, 5, 10, and 20 μM concentrations were performed in reaction conditions containing 50 mM Hepes (pH 8.0), 50 mM KCl, 5% glycerol, and 1 mM TCEP and were initiated with 100 nM nucleosome substrate. Aliquots of 10 μl were withdrawn at t = 0, 2, 5, 30, and 120 min time points and quenched using Laemmli dye followed by boiling for 2 min to halt demethylation. Half of the time-course reaction mixture was applied to conditions with a H3K4me2 specific antibody (EMD Millipore, #07-030, LOT: 3920776) on one blot, where antibody specificity was verified by vendors and in the lab. The other half of the reaction mixture was incubated with an H3 antibody (Abcam #EPR16987), with specificity verified by Abcam and within the lab, in a separate blot. The assay products were resolved by 20% SDS-PAGE gel for 90 min at 200 V, and then transferred to immunoblot polyvinylidene difluoride membranes and blocked with 4% fat free milk in PBS buffer. Blots were H3K4me2 specific antibody incubated with H3K4me2 or H3 specific antibodies overnight followed by goat anti-rabbit antibody for 1 h. The blot images were visualized by chemiluminescence, and all *in vitro* WB data were analyzed using GE Amersham Imager 600 software. Demethylation and control experiments were performed in duplicate or triplicate (N = 2 or 3) and careful analysis and quantification of the kinetic data was performed in a similar manner as previously described (39, 49). Quantitation of each data point was obtained by dividing the H3K4me2 signal by the H3 antibody signal and the H3K4me2/H3 ratio was plotted as a function of time (minutes) and subsequently analyzed using nonlinear regression (GraphPad Prism 8).

For examining the effect of NLS peptides on LSD1 catalyzed demethylation activity, different concentrations of NLS (0, 10 μM, 20 μM, 50 μM, 100 μM, and 400 μM) or NLSp∗ (0, 10 μM, 40 μM, and 100 μM) were incubated with the 2 μM ΔN LSD1-CoREST containing reaction mixture in the presence of the purified nucleosome substrate (EpiCypher, 16–1334), at 25 °C for 0, 2, 5, 30, and 120 min. Here, all demethylation reaction mixtures contained 2 μM ΔN LSD1-CoREST and the buffer 50 mM Hepes (pH 8.0), 50 mM KCl, 5% glycerol, 1 mM TCEP, and were initiated by adding 100 nM nucleosome substrate as previously described (49). Quantitative data were fit to the equation $\left[H3K4\text{me}2\right]={\left[H3K4\text{me}2\right]}_{t=0}e^{–\text{kobs}*t}$ and the relative rate constants (Kobs) were evaluated at increasing NLS or NLSp∗ concentrations. The extraction of the estimated IC_50_ parameters for the NLS and NLSp∗ peptides were determined as previously described (49), with an assumption of 1:1 LSD1: peptide binding. Control experiments were performed for each of the conditions above with the aforementioned buffer conditions with 100 μM NLS in the absence of ΔN LSD1- CoREST.

### CD, NMR spectroscopy, and X-ray crystallography

CD measurements of the NT-LSD1 (aa 100–151) and the NLS peptide (aa 107–120) were recorded with a Chirascan V100 CD spectrophotometer equipped with Peltier temperature control and calibrated with d-10-camphorsulfonic acid. Wavelength scans between 190 to 260 nm were carried out at 20 °C with a 1 mm cell, 1 nm bandwidth, 4 s per point, and 0.1 ms timed intervals. Spectra from a NT-LSD1 protein at 10 μM or NLS peptide at 15 μM were both recorded in 10 mM sodium phosphate (pH 8.0), 0.1 M NaF were averaged over three scans, and background from a buffer only matched sample was subtracted. Data for wavelength scans are presented in units of molar ellipticity ([θ], deg cm^2^ dmol^−1^) or millidegrees (mdeg).

All NMR experiments were conducted under the conditions of 10 mM Hepes pH 6.8, 50 mM KCl, and 50 μM 4, 4-dimethyl-4-silapentane-sulfonate at 298 K with 10% (v/v) D_2_O. NMR spectra were acquired on Bruker Avance-III 600 MHz, Varian Inova 600 MHz and Varian 800 MHz spectrometers, equipped with cryogenic probes. A set of traditional triple-resonance experiments were performed on a 0.8 mM ^15^N,^13^C-labeled N-terminus peptide LSD1 sample, including HNCO, HNCA, HN(CO)CA, HNCACB, CBCA(CO)NH, HN(CA)CO, and H(CCO)NH (36, 50, 51, 52, 53) For intermolecular NOE extraction, a three-dimensional 15N-NOESY-HSQC (54) was executed on a 0.3 mM ^15^N-labeled NT-LSD1 sample (residues 100–151). ^1^H chemical shifts were referenced with respect to internal 4, 4-dimethyl-4-silapentane-sulfonate, and ^13^C and ^15^N chemical shifts were referenced indirectly using nuclei-specific gyromagnetic ratios (55). Spectra were processed with Topspin 3.5.7 (Bruker Inc., https://www.bruker.com/) and NMRPipe (56), and analyzed using SPARKY (57) (https://nmrbox.nmrhub.org/). Secondary structural elements of NT-LSD1 were predicted by importing chemical shifts to the Talos-N web server (35). Excluding the N-terminal nonnative glycine and serine, 48 of 51 nonproline residues were assigned in the (^1^H, ^15^N) HSQC spectrum, which corresponds to 94.1% of the backbone amides (Figs 3*B* and S6, *B* and *C*, BMRB 27615).

Crystals of the truncated ΔN LSD1-CoREST, diffraction data (3.2–3.5 Å) were obtained as previously described and X-ray diffraction data were collected at LS-CAT-21 (21-G) at Argonne National Laboratory. Data were processed and scaled using the autoPROC (58). Molecular replacement was applied to locate a solution using PHENIX (https://phenix-online.org) with a previously determined structure of LSD1-CoREST (PDB 4XBF) (14, 59). For the peptide soaked LSD1-CoREST crystals, all diffraction data were collected at LS-CAT (21-G). A molecular replacement solution was obtained and was applied using Phaser (60). After various crystal soak trials, the difference Fourier map (|*F*_*o*_|-*|F*_*c*_|) identified the location of the NLS peptide within the LSD1-CoREST crystals and model building of the peptide into the difference density was performed in Coot (61).

### Structure prediction and MD simulation for the NT-LSD1 (100–151) or NT-helix (135–151)

Rosetta models generated using NMR chemical shifts were performed as described elsewhere (62). Briefly, the Talos + server was used to predict secondary structure information for the NT-helix (135–151) and the NT-LSD1 (100–151) regions using chemical shift data from NSQC, HNCACB, and CBCA(CO)NH (63). Fragments were selected on the basis of these secondary structure predictions for *de novo* structure prediction. Initially, 40,000 models were generated, and the best scoring 10% of models were clustered with Calibur (64). A total of 10,000 additional models were generated starting from the best scoring model in each of the largest three clusters to make a total of 70,000 models. Simultaneously, the CS-Rosetta server was used to generate 40,000 models from HNCO, HNCA, HN(CO)CA, HNCACB, CBCA(CO)NH, and HN(CA)CO NMR data (62), and only the lowest energy models revealed an α-helical structure (aa 135–148) consistent with NMR data.

The 52 residue N-terminal region of LSD1 (amino acid residues 100–151) and the NT-helix (135–151) were also subject to *de novo* structure prediction with Rosetta (65, 66, 67, 68). Briefly, fragments were generated with the Robetta online server (69) and used to construct models with the Rosetta *ab initio* application. Subsequently, 25,000 models were generated for both polypeptides, with the best scoring models in each of the largest clusters comprising a total of 75,000 models. The top 10% of all models were then clustered with Calibur (64). A comparison of the *Ab initio* and CS-Rosetta approaches for each model was calculated with respect to the best scoring model, and all models were classified according to the Talaris2014 score energy function (70) (Table S3).

MD simulations of the NT-LSD1 (100–151) or (135–151) were also performed to gain insight into the stability of the N-terminal α-helix. Briefly, all models were allowed to equilibrate in a rectangular box of TIP4PEW explicit solvent neutralized with monovalent sodium (71). Solute was buffered on all sides with a 10 Å solvent. An all-atom minimization occurred in three stages: First, the solvent was minimized around the restrained protein with 5000 steps of steepest gradient descent followed by 10,000 steps of conjugate gradient descent. Second, the protein was minimized with ENREF24 (72) in restrained buffer for 2000 steps steepest gradient descent followed by 8000 steps of conjugate gradient descent. Restraints in both stages were weighted at 10 kcal/mol. Finally, 500 steps of steepest gradient descent following by 9500 steps of conjugate gradient descent in unrestrained minimization of the entire system were performed. Post minimization (SHAKE) was implemented to constrain covalent bonds to hydrogen atoms (73), and systems were slowly heated in NVT ensemble to 100K over 50 ps with a 1 fs timestep. The system was then heated in NPT ensemble at 1 bar with isotropic position scaling from 100K-300K over 500 ps and a 1 fs timestep. The equilibration and production simulations were performed in NPT ensemble at 300K for 2 ms or 10 ms with a Monte Carlo barostat and 2 fs timestep, and temperature was controlled using Langevin dynamics with a collision frequency of 1 ps-1 and a random seed for each independent simulation (74, 75). Two independent equilibration and production simulations above were performed starting from the lowest energy structures from each of the top three clusters generated from Rosetta *ab initio* and two of the best scoring models from CS-Rosetta predictions (Table S2).

### MD simulation of the H3 (aa 3–11) and NLS (aa 109–118) peptides at LSD1’s active site cleft

For both H3 and NLS peptides and the LSD1-CoREST structure (PDB 6VYP), the GPU version of AMBER with PMEMD implementation of SANDER was used (76, 77), using the ff19SB force field to model interactions (78), and the optimal point charge force field to describe water properties (79). The protein with the peptides (system) was solvated within a cubic periodic box with water molecules using the LEAP module, and counter ions were added to achieve physiological salt concentrations (0.15 M). Solute was buffered on all sides with 12 Å solvent, and the system and solvent were minimized separately to alleviate steric clashes in a similar manner as defined for the NT-LSD1 system with the following modifications. The equilibration process involved heating the system 0 to 100 K using a Langevin thermostat (ntt = 3) with a collision frequency of 5 ps^−1^ to emulate thermal fluctuations. The system was then heated to 300 K as the periodic boundaries were imposed throughout, followed by equilibration over 250 ps with a 4 fs timestep and under constant pressure (1 bar) conditions to ensure proper density fluctuations and system stability. The production simulation was then conducted under the NPT ensemble, maintaining constant temperature (300 K) and pressure (1 bar) using Langevin dynamics with a random seed for each independent simulation. Integration of thermal motion was performed, and 10 trajectories of 2 μs were post processed and analyzed using the CPPTRAJ feature in AMBER. Long-range electrostatic interactions were handled using the Particle Mesh Ewald method (76), and the trajectory files were analyzed using VMD software (https://www.ks.uiuc.edu) (80), Here, a subset of 10 frames, with a time step of 200 ns was extracted from the trajectory for further analysis, corresponding RMSD calculations at 0.2, 0.4, 0.6, 0.8, 1.0, 1.2, 1.4, 1.6, 1.8, and 2 ms time points for the H3 and NLS–based simulations (Fig. S12*C*). Frame alignment and RMSD calculations were conducted in Pymol (https://www.pymol.org), utilizing the reference MD starting structure, which was derived from the H3-LSD1 (6VYP) or the difference density NLS docked-LSD1 model (Fig. S12*A*) (80, 81).

## Data availability

NMR chemical shift data are available at BMRB (27615). Any additional information, plasmids, reagents, and data are available from the corresponding author at nicholas.reiter@marquette.edu upon request.

## Supporting information

This article contains supporting information (9, 12, 29, 68, 69, 82, 83, 84, 85).

## Conflict of interest

The authors declare that they have no conflicts of interest with the contents of this article.

## Supporting information

Supplemental_Movie_SM1
