# Supplementary material for: Autoregulatory mechanism of enzyme activity by the nuclear localization signal of lysine-specific demethylase 1[image]
Source: J Biol Chem. 2024 Jul 30;300(9):107607. doi: 10.1016/j.jbc.2024.107607 (PMC11388019; doi:10.1016/j.jbc.2024.107607)
Supplement: Supplemental_Movie_SM1 [file mmc1.pptx]

## Slide 1
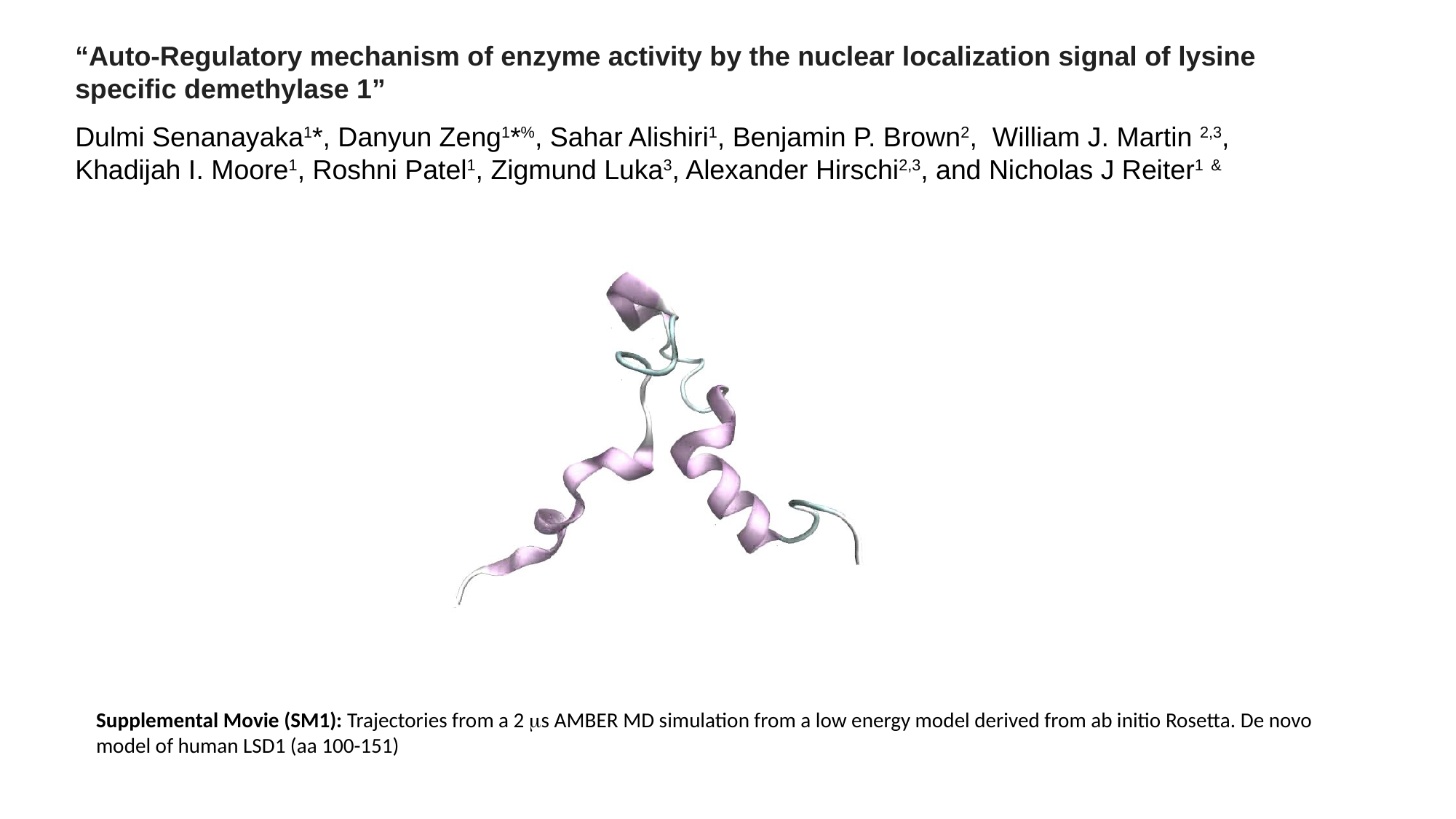

“Auto-Regulatory mechanism of enzyme activity by the nuclear localization signal of lysine specific demethylase 1”
Dulmi Senanayaka1*, Danyun Zeng1*%, Sahar Alishiri1, Benjamin P. Brown2, William J. Martin 2,3, Khadijah I. Moore1, Roshni Patel1, Zigmund Luka3, Alexander Hirschi2,3, and Nicholas J Reiter1 &
Supplemental Movie (SM1): Trajectories from a 2 ms AMBER MD simulation from a low energy model derived from ab initio Rosetta. De novo model of human LSD1 (aa 100-151)
