# Supplementary material for: Autoregulatory mechanism of enzyme activity by the nuclear localization signal of lysine-specific demethylase 1[image]
Source: J Biol Chem. 2024 Jul 30;300(9):107607. doi: 10.1016/j.jbc.2024.107607 (PMC11388019; doi:10.1016/j.jbc.2024.107607)
Supplement: Supporting Figures and Tables [file mmc2.pdf]

SUPPORTING INFORMATION

Auto-Regulatory mechanism of enzyme activity by the nuclear localization signal of lysine specific demethylase 1

Dulmi Senanayaka[1*], Danyun Zeng[1*%], Sahar Alishiri[1], William J. Martin [2,3], Khadijah I. Moore[1], Roshni Patel[1], Zigmund Luka[3], Alexander Hirschi[2,3], and Nicholas J Reiter[1 &]

* Indicates equal contribution

[1] Department of Chemistry, Marquette University, Milwaukee, WI USA

[2] Center for Structural Biology, Vanderbilt University School of Medicine, Nashville, TN USA

[3] Department of Biochemistry, Vanderbilt University School of Medicine, Nashville, TN USA

[&]Corresponding author: Nicholas J Reiter

Email: nicholas.reiter@marquette.edu

Contact Information: Nicholas Reiter, Marquette University, 1414 W. Clybourn St., Milwaukee WI, 53233

[%] **Present Address:** Wuhan Institute of Physics and Mathematics, Innovation Academy for Precision Measurement Science and Technology, Chinese Academy of Sciences, Wuhan 430071, China

Supporting Table S1. Peptide fragments tested in the present study. The nuclear localization signal (NLS) regions (light blue) and amino acid modifications (red) are indicated, with numbering corresponding to human LSD1 (KDM1A). With exception of NT-LSD1, all peptides contain acetylation and amidation modifications at the N- and C-termini, respectively.

LSD1

**Name**	**Peptide region**
NLS-LSD1 (NLS)	^{107}GRRTS**RRKRAK**VEY^{120}
Nt LSD1 P1 (P1)	^{121}REMDESLANLSEDEYY^{136}
Nt LSD1 P2 (P2)	^{137}SEEERNAKAEKEKKL^{151}
NLS-LSD1 phosphomimic (NLSp*)	^{107}GRR**DD****RRKRAK**VEY^{120}
NLS-LSD1 K114me (NLS-K114me2)	^{107}GRRDD**RRK****me2****RAK**VEY^{120}
N-terminus peptide (NT-LSD1)	^{100}GIAETPEGRRTSRRKRAKVEYREMDESLANLSEDEYYSEEERNAKAEKEKKL^{151}

H3

**Name**	**Peptide region**
H3	^{1}ARTK**me2**QTARKSTGGKAPRKQLA^{21}

Supporting Table S2. Summary of molecular dynamics simulation times Two independent equilibration/production simulations were performed starting from the lowest energy structure from each of the top three clusters generated by Rosetta *ab initio*. The top ten best scoring structures from CS-Rosetta predictions of human LSD1 residues 135-151 and human LSD1 residues 100-151 were all simulated in independent trajectories. All independent equilibration/production simulations were 2.0 µs in duration. [1-3]

Modeling Method	Protein Model	Total Production Simulation Time (µs)	# Independent Trajectories
Rosetta *ab initio*	human LSD1 residues 100-151 cluster 1	2.0	2
Rosetta *ab initio*	human LSD1 residues 100-151 cluster 2	2.0	2
Rosetta *ab initio*	human LSD1 residues 100-151 cluster 3	2.0	2
CS-Rosetta	human LSD1 residues 135-151	10.0	10
CS-Rosetta	human LSD1 residues 100-151	10.0	10
		26.0	26

Supporting Table S3. Score function comparison of rosetta Ab initio vs. CS-Rosetta approaches for the ten lowest energy LSD1 N-terminal domain models.

Abinitio		CS		Abinitio-->Abinitio		Abinitio-->CS		CS-->CS		CS-->Abinitio	
SCORE	ID	SCORE	ID	SCORE	ID	SCORE	ID	SCORE	ID	SCORE	ID
-92.128	S_00000538_2	-83.349	S_00000093_3	-87.723	S_00000611_1	-79.102	S_00000710_4	-79.011	S_00000424_3	-88.099	S_00000719_2
-89.698	S_00000817_1	-79.517	S_00001408_1	-84.230	S_00000349_2	-77.484	S_00000804_1	-77.865	S_00000341_1	-86.363	S_00000361
-89.021	S_00001390_1	-79.445	S_00000346_6	-84.176	S_00000889_4	-75.257	S_00000952_2	-77.477	S_00000884_0	-86.293	S_00000896_3
-88.372	S_00001627_4	-78.194	S_00000695_1	-83.811	S_00000737_0	-74.891	S_00000841_1	-77.221	S_00000115_1	-85.998	S_00000433_3
-88.240	S_00001079_2	-78.000	S_00000374_6	-83.704	S_00000485_0	-74.852	S_00000464_1	-76.716	S_00000953_0	-85.303	S_00000062_3
-87.938	S_00000627_4	-77.980	S_00000399_1	-83.459	S_00000644_4	-74.333	S_00000063_0	-76.546	S_00000231_0	-84.447	S_00000830_4
-87.508	S_00001397_3	-77.964	S_00000391_0	-83.437	S_00000790_3	-74.116	S_00000709_3	-75.989	S_00000809_4	-84.404	S_00000324_1
-87.459	S_00000674_8	-77.777	S_00000901_6	-83.369	S_00000368_2	-73.947	S_00000926_1	-75.378	S_00000449_4	-84.065	S_00000593_1
-87.351	S_00001809_9	-77.408	S_00001840_2	-82.870	S_00000832_1	-73.896	S_00000904_1	-75.001	S_00000315_0	-83.996	S_00000238_4
-86.257	S_00000612_9	-76.967	S_00000524_3	-82.793	S_00000178_1	-73.854	S_00000651_1	-74.860	S_00000872_3	-83.860	S_00000657_4

Supporting Table S4. Comparative (K_i ^{app}) data of peptide sequences that inhibit LSD1-catalytzed demethylation on a H3K4me2 model (peptide) substrate.

No	Peptide	Sequence	K_i/μM
1	NLS (14 residues)	GRRTSRRKRAKVEY	3.3
2	NLSpp-mimic		
3	NLS-K114me2	GRRDDRRKRAKVEY	38.7
		GRRDDRRKme2RAKVEY	-, ND&
4	[a] SNAIL (1-20)	PRSFLVRKPSDPNRKPNYSE	0.21
5	[a] INSM1 (1−20)	PRGFLVKRSKKSTPVSYRVR	0.24
6	[b] H3 (1-21)	ARTKQTARKSTGGKAPRKQLA	1.8
7	[b] H3 (1−12)	ARTKQTARKSTG	199
8	[d] H3 (1-21) K4M	ARTMQTARKSTGGKAPRKQLA	0.05
9	[c] H3(1-21)K4meR8me	ARTKmeQTARmeKSTGGKAPRKQLA	100
10	[c] H3(1-21)K4me3	ARTKme3QTARKSTGGKAPRKQLA	19.5
11	[c] H3(1-21) K4R	ARTRQTARKSTGGKAPRKQLA	0.41
12	[b] H3(5-21)	QTARKSTGGKAPRKQLA	87
13	[b] H3(1-21) K4me S10p	ARTKmeQTARKSpTGGKAPRKQLA	31
14	[e] H3(1-21) $K4^{propargylamine}$	$ARTK^{prop}$QTARKSTGGKAPRKQLA	16.6

All peptides, except (14), contain C-terminal amides. Unless denoted (&), all peptides exhibit competitive inhibition.

[a] Data from reference [4]
[b] Data from reference [5, 6]
[c] Data from reference [7]
[d] Data from reference [8]
[e] Data from reference [9]

Supporting Figures.

SFigure 1.

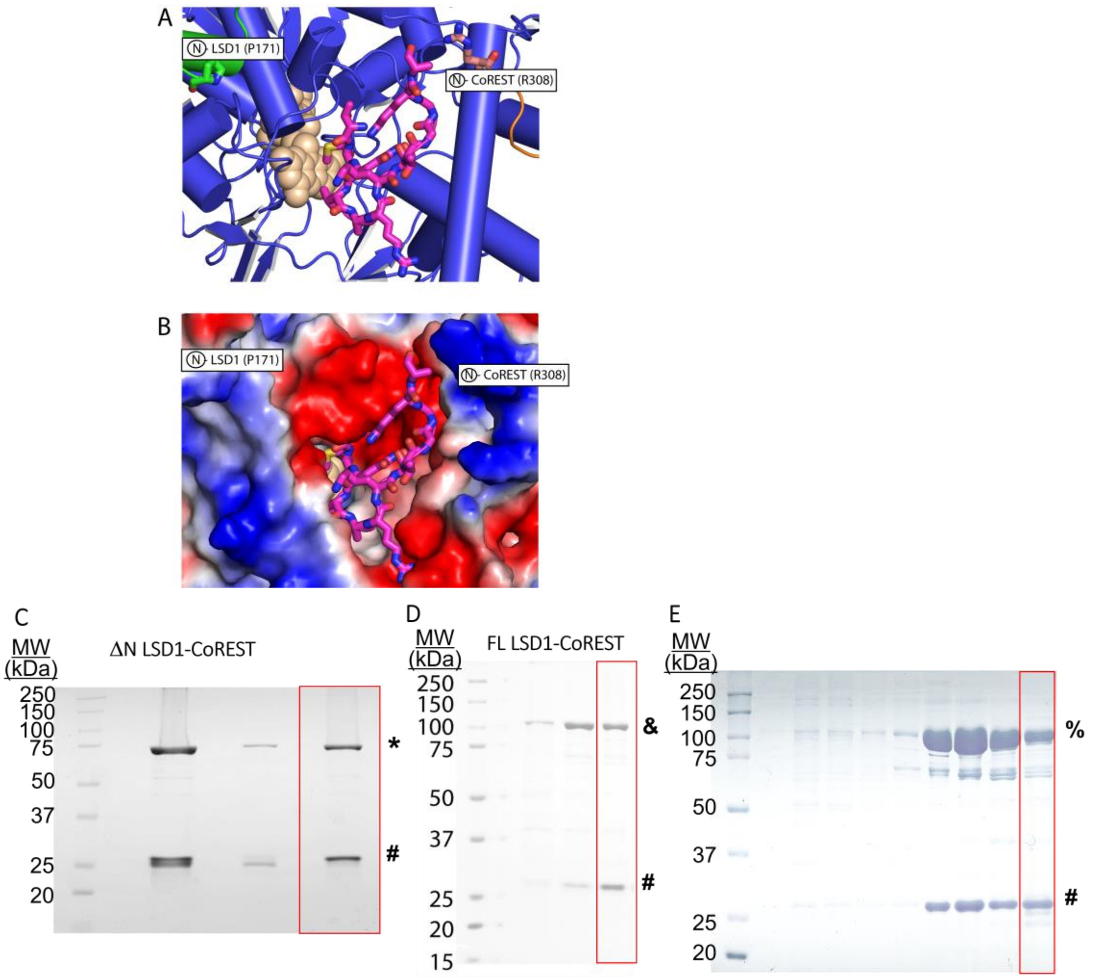

Supporting Figure S1. Active site region of LSD1 in complex with the H3 tail from the LSD1-nucleosome structure (PDB 6VYP) and purity gels of LSD1-based constructs used in this study. A) Ribbon representation of the active site reveals the N-termini of LSD1 (green, P171) and CoREST (orange, R308) in close proximity to the active site and H3 peptide (magenta, sticks). LSD1 is blue, the SWIRM domain of LSD1 is green, and the cofactor FAD (beige) is shown. **B)** Electrostatic structure of the active site region of LSD1 in the identical orientation as **A)**. The N-terminus extends from the SWIRM domain (green, residue 171) of LSD1 and is positioned towards the active site cavity, whereas the C-terminus extends from CoREST (residue 308 in the structure). Both regions sit atop the catalytic pocket and near the extranucleosomal DNA. The LSD1-CoREST-nucleosome structure (PDB 6VYP) suggest that disordered regions (N-termini of LSD1, CoREST) are in

the vicinity of the enzyme active site and play a role in positioning and regulating access to the substrate. **C-E)** 10% SDS-PAGE gel of LSD1-CoREST complexes after subjected to size-exclusion chromatin (SEC). Red boxes denote fractions used for biochemical studies and the molecular weight marker (MW) given in kiloDaltons (kDa) is provided in each gel . **C)** Complexes of purified ΔN LSD1 (171-852) – CoREST (286-482) located at asterisk and hashtag, respectively, **D)** purified full length LSD1 (1-852) – CoREST (286-482) located at ampersand and hashtag, respectively, and **E)** purified full length (T110D/S111D) LSD1 – CoREST (286-482) located at percentage and hashtag, respectively, are noted.

SFigure 2.

A

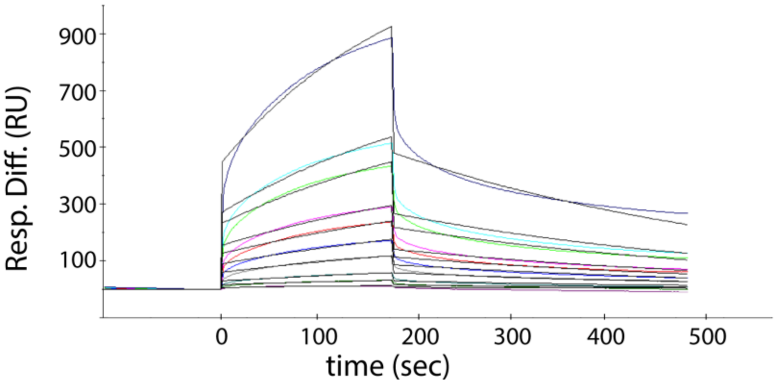

B

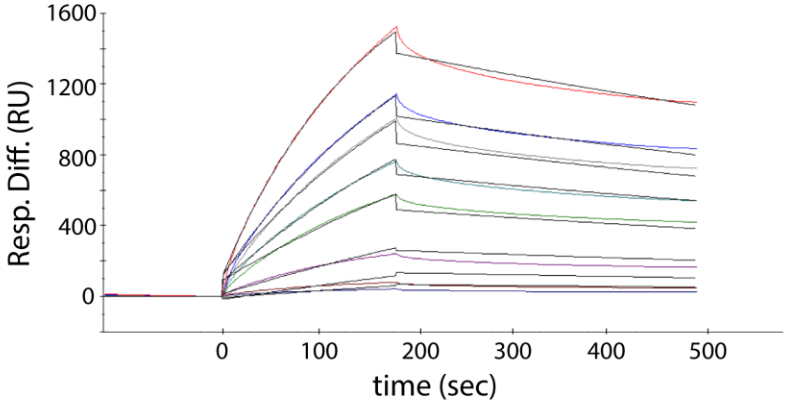

Supporting Figure S2. SPR fitted data analysis for **A)** ΔN LSD1-CoREST and **B)** full-length LSD1-CoREST to immobilized nucleosomes. Immobilized nucleosomes were subject to increasing concentrations of the LSD1-CoREST complex. In **A**) and **B)**, raw sensorgram data (colored) is shown for 5 nM (dark blue), 10 nM (blue) 25 nM (dark red), 50 nM (magenta), 100 nM (pink), 200 nM (orange), 300 nM (turquoise), 400 nM (cyan), 500 nM (light orange), 2 μM (green) LSD1-CoREST concentrations**.** Curve fitted data analysis from Biacore's BIAevaluation software 3.0 was performed for each response units (RU) trace at each ligand concentration. This enabled an apparent dissociation constant (K_d) for the nucleosome-LSD1-CoREST interaction.

SFigure 3.

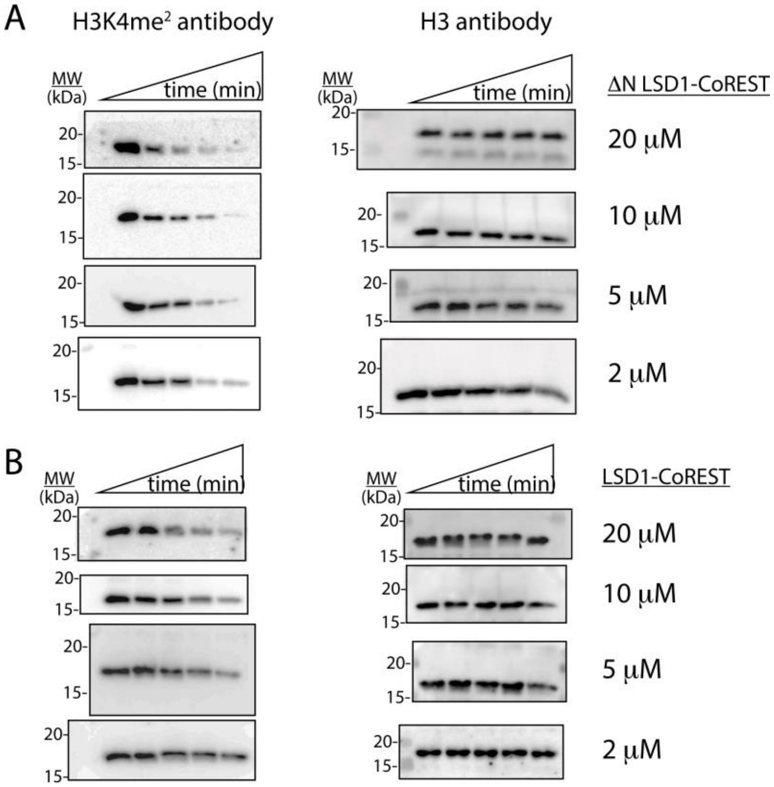

Supporting Figure S3: Representative western blots monitoring H3K4me2 mononucleosome demethylation at varying concentrations of A) ΔN LSD1-CoREST and B) full-length LSD1-CoREST. Western Blots were performed after a time course reaction of LSD1 demethylation on H3K4me2 nucleosomes. All demethylation reaction mixtures contained either 2, 5, 10, or 20 μM LSD1- CoREST, were performed in the buffer 50 mM HEPES (pH 8.0), 50 mM KCl, 5% glycerol, 1 mM TCEP, and were initiated by adding 100 nM nucleosome substrate into the reaction mixture. Aliquots of exactly 10 μL were withdrawn at each time point (t= 0, 2, 5, 30, 120 minute time points) and quenched using Laemmli dye followed by boiling for 2 min to halt the demethylation reaction. The assay products were resolved by 20% SDS-PAGE gel for 90 minutes at 200 V. The degree of inhibition was measured using H3K4me2 specific antibody (at left for **A** and **B**) relative to the amount of H3 in each lane using H3 specific antibody (at right for **A** and **B**). Thus, half of the extracted 10 μL reaction mixture was incubated with a H3K4me2 specific antibody on one blot and the other half of the reaction mixture was incubated with a H3 specific antibody in a separate blot. The activity assay and the image analysis were performed in duplicate for each condition and the densitometry data was visualized by chemiluminescence using Amersham software. Image disclosure: Figure S3A (20 and 2 μM experiments) are redundant with Figure 2C and Figure S3B (20 and 2 μM experiments) are redundant with Figure 2D. Image Justification:

Images are shown together in the context of the entire western blot assay series (N = 2). Analysis of the data and all time course experiments enabled the quantification of LSD1 activity on H3K4me2 nucleosome substrates *in vitro*.

SFigure 4

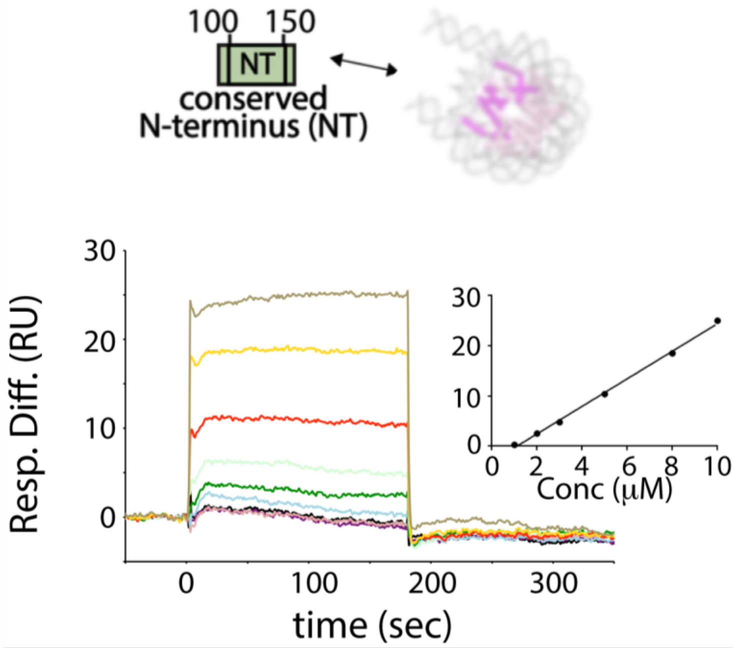

Supporting Figure S4. The NT-LSD1 non-specifically interacts to nucleosomes weakly. The weak binding by the conserved N-terminus (residues 100-151) appears to minimally contribute to the observed increased binding of the FL-LSD1 to the nucleosome (Figure 2B). A biotinylated nucleosome was immobilized on an SA sensor chip and was subject to 100 nM (pink), 500 nM (black), 1 μM (light blue), 2 μM (green), 3 μM (light green), 5 μM (red), 8 μM(yellow), and 10 μM (beige) NT-LSD1 concentrations. Raw surface plasmon resonance sensorgram data (N=2) and an analysis of Response Units (RU) versus NT-LSD1 ligand concentration (inset) suggest very weak, non-specific binding interactions.

SFigure 5

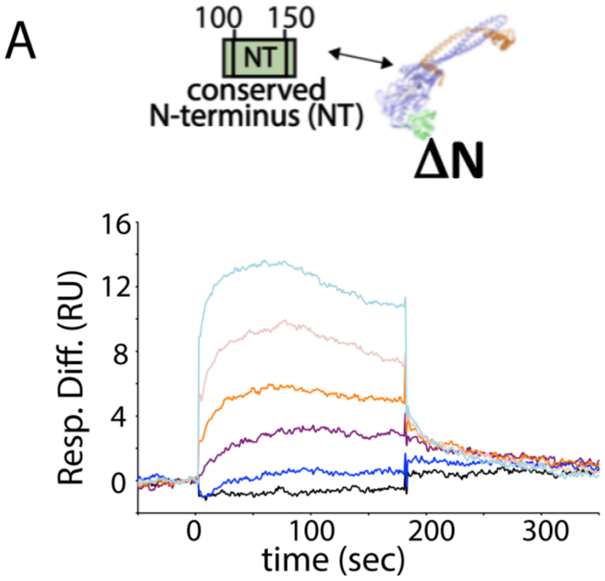

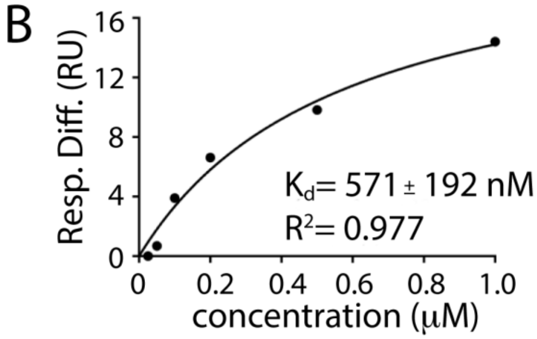

Supporting Figure S5. Binding interaction of the NT-LSD1 to ΔN LSD1-CoREST. A,C) Surface Plasmon Resonance data (A) shows the differential response units (RU) over time, measuring how the LSD1-coREST complex interacts with the NT-LSD1 that is immobilized on an SA sensor chip. The NT-LSD1 was subject to 5 nM (black), 25 nM (blue), 50 nM (purple), 100 nM (orange), 200 nM (light pink), and 300 nM (cyan) protein concentrations. B) Analysis of the different in RU versus LSD1-CoREST concentration reveals the binding dissociation constant (K_d) for the binding interaction. *Image disclosure*: Figure 3A is redundant with Supporting Figure S5B above. *Justification*: Figure 3A represents the analysis of raw sensorgram data in Support Figure S5A. Reuse of Support Figure S5B thereby consolidates all source data associated with this specific series of SPR experiments.

SFigure 6

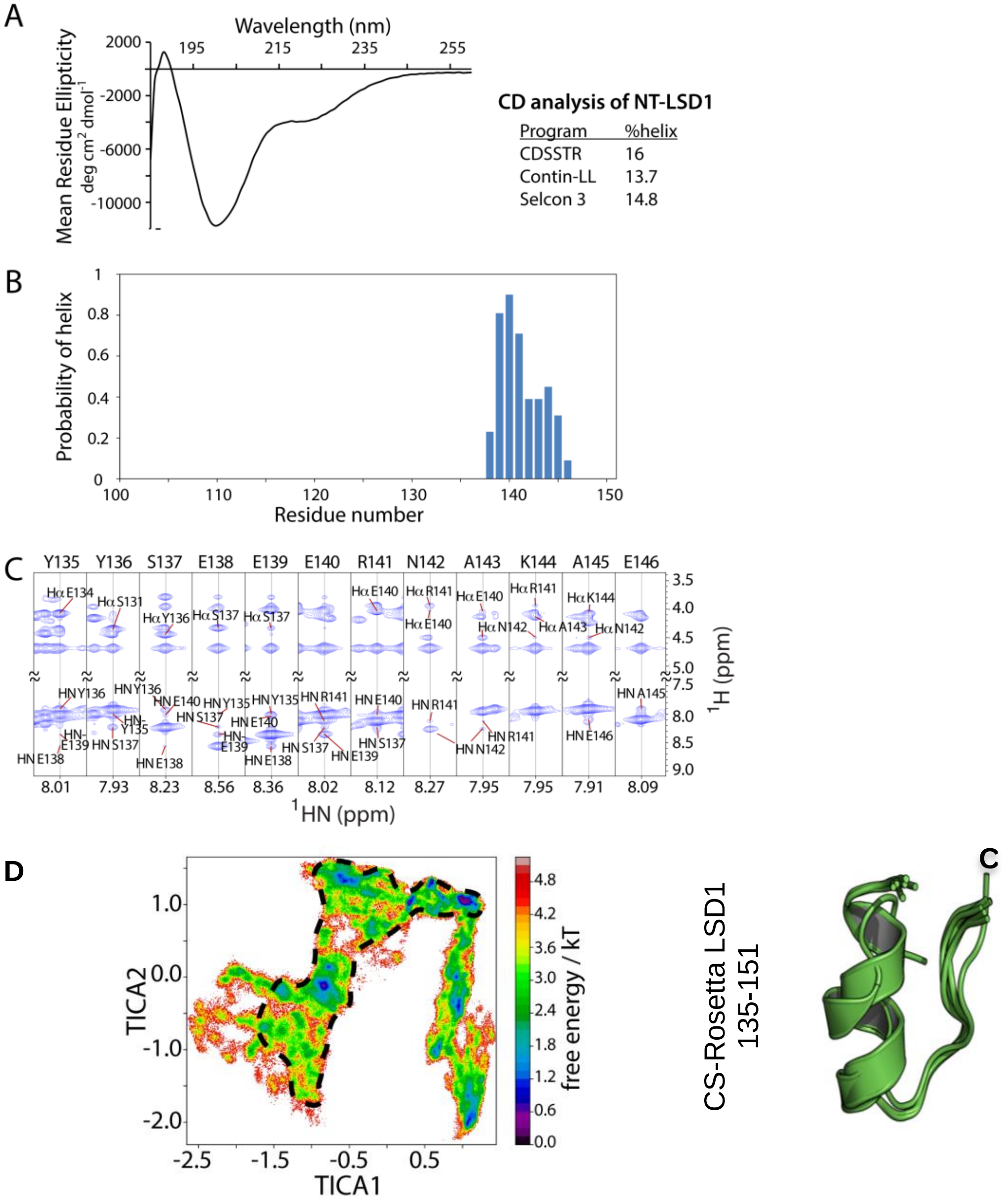

A
Wavelength (nm)
195
215
235
255
2000
-2000
-6000
-10000
Mean Residue Ellipticity
deg cm^2 dmol^{-1}
CD analysis of NT-LSD1
Program %helix
CDSSTR 16
Contin-LL 13.7
Selcon 3 14.8
B
Probability of helix
Residue number
0
0.2
0.4
0.6
0.8
1
100
110
120
130
140
150
C
Y135 Y136 S137 E138 E139 E140 R141 N142 A143 K144 A145 E146
8.01 7.93 8.23 8.56 8.36 8.02 8.12 8.27 7.95 7.95 7.91 8.09
^{1}HN (ppm)
^{1}H (ppm)
3.5
4.0
4.5
5.0
7.5
8.0
8.5
9.0
D
TICA2
TICA1
-2.5
-1.5
-0.5
0.5
1.0
0.0
-1.0
-2.0
free energy / kT
4.8
4.2
3.6
3.0
2.4
1.8
1.2
0.6
0.0
CS-Rosetta LSD1
135-151
C

E

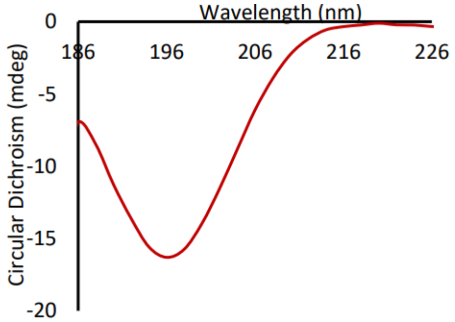

CD spectroscopy of the NLS peptide (aa 107-120)

Supporting Figure S6. Spectroscopic structural characterization of the NT-LSD1 (100-151). A) CD measurements were obtained with a obtained with a Jasco J-810 spectropolarimeter equipped with a Peltier temperature control device and calibrated with d-10-camphorsulfonic acid. The data pitch was 1 nm, the scan speed 20 nm/min, the response time 5 s, and the band width 1 nm, and spectrum is presented in units of mean residue ellipticity ([θ]), deg?cm2?dmol-1, focusing on far-UV regions that give rise to secondary structure. Three independent CD analysis programs identify the estimated α-helical content of NT-LSD1. **B)** TALOS-N web server output. Secondary structural elements were predicted by importing chemical shifts (BMRB 27615) to the TALOS-N web server. Excluding the N-terminal non-native glycine and serine, 48 of 51 non-proline residues were assigned in the (^{1}H, ^{15}N) HSQC spectrum, which correspond to 94.1 % of the backbone amides (Figure 3B). **C)** ^{15}N Nuclear Overhauser Effect (NOE) sections (mixing time, 250 ms) of the NT-LSD1 region showing inter- and intramolecular NOE connectives that are indicative of formation of an α-helix. Spectrum parameters are described in the experimental section. **D)** Time independent component analysis (tICA) was derived from independent 2.5 μs Amber simulations using the a99SB-disp forcefield and initial models from CS-Rosetta/Rosetta predictions. Three groupings of structures were predicted based upon this analysis, suggesting substantial structural diversity and timescales of conformational transitions across the NT-LSD1 structure. 1) A group of conformations with a helical structure (aa 135-148) with all other regions (100-134, 149-151) displaying high disorder (dashed box region) was observed, 2) conformations that are completely disordered are present (outside boxed area), and 3) the probability of a helical turn segment (128-135) occuring in the center of the polypeptide forming with or without the helical structure (aa 135-148) was also observed (see supplemental Movie 1). A plot is color-coded according to the free energy/kT and this tICA plot is supportive of the NMR NOE data analysis in C. At right are 10 low-energy structure predictions of the aa135-148 region based upon NMR data and CS-Rosetta. **E)** CD measurements between 185-228 nm of an 5 μM NLS peptide (107-120) recorded at room temperature and obtained on a Chirascan V-100 spectropolarimeter. The data pitch was 1 nm, the scan speed 20 nm/min, the response time 5 s, and the band width 1 nm, and spectrum is presented in units of ellipticity ([θ]), mdeg, focusing on far-UV regions that give rise to secondary structure. This NLS peptide contains no structure and exhibits intrinsically disordered characteristics.

SFigure 7

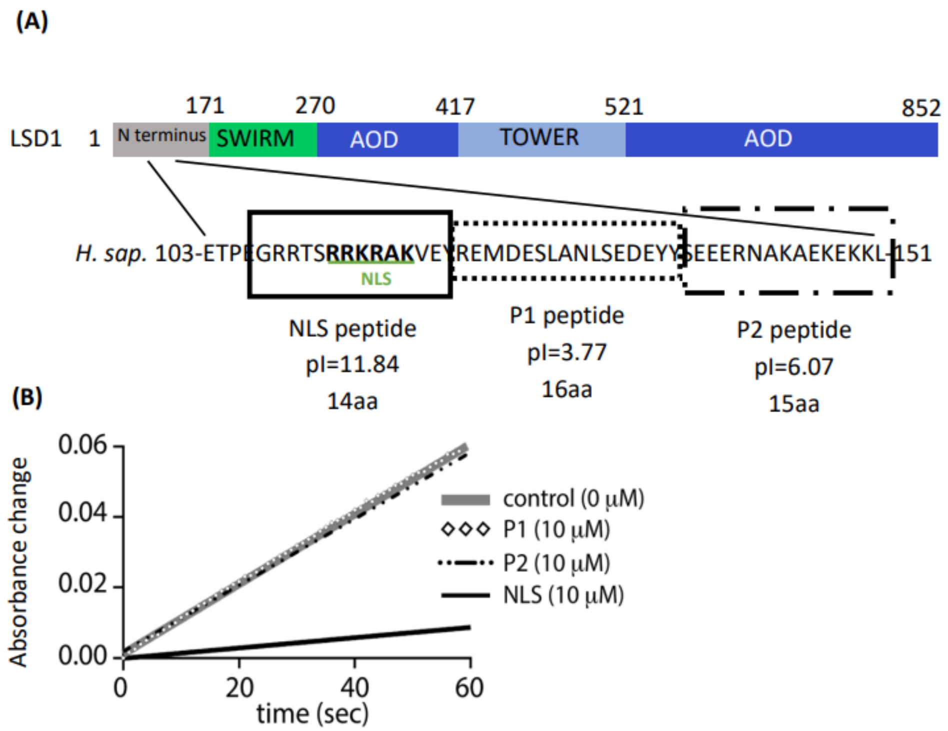

Supporting Figure S7. The conserved region of the LSD1 N terminus contains an NLS sequence that inhibits LSD1 activity. A) Domain organization of LSD1 and sequence of conserved regions of the LSD1 N terminus (*H. sap.*). Sequences termed: NLS, P1, and P2 represent peptide regions that were examined and are illustrated in boxes with the corresponding isoelectric (PI) values. **B)** Plot of absorbance change versus time (sec) using an LSD1 - horseradish peroxidase coupled assay. The NLS peptide (black line) inhibits LSD1 activity on a H3K4me2 model peptide substrates while peptides P1 and P2 (dashed and dash-line-dash, respectively) do not inhibit LSD1's activity. For the NLS, an approximate Ki was determined through measuring the initial velocity versus H3K4me2 21 a.a. peptide substrate concentration (see Figure 4A).

SFigure 8

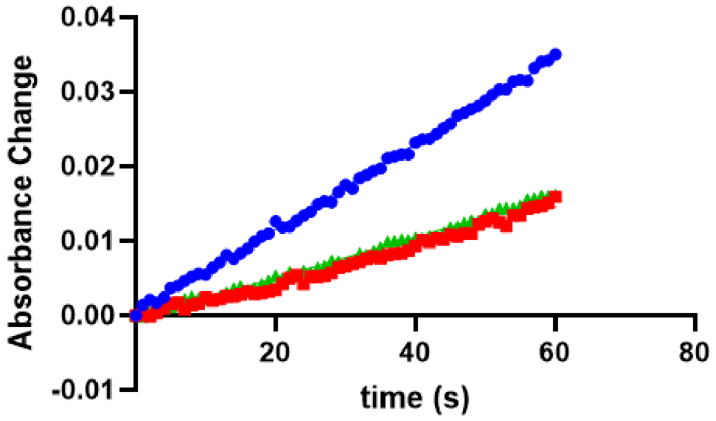

Supporting Figure S8. Peptide-based inhibition is independent of Horseradish peroxidase (HRP) concentration in the coupled demethylase reaction. Control experiment to assess the influence of the HRP concentration on 20 μM [H3K4me2 peptide substrate]. The LSD1-demethylase time course reaction at 0 μM inhibitor (blue), 5 μM NLS-peptide and 1X HRP (1 μg/150 μL) (red), and with 5 μM inhibitor and 2X HRP (2 μg/150 μL) (green). No change in activity due to HRP changes indicates the NLS peptide directly influences the LSD1 catalyzed demethylation reaction.

SFigure 9

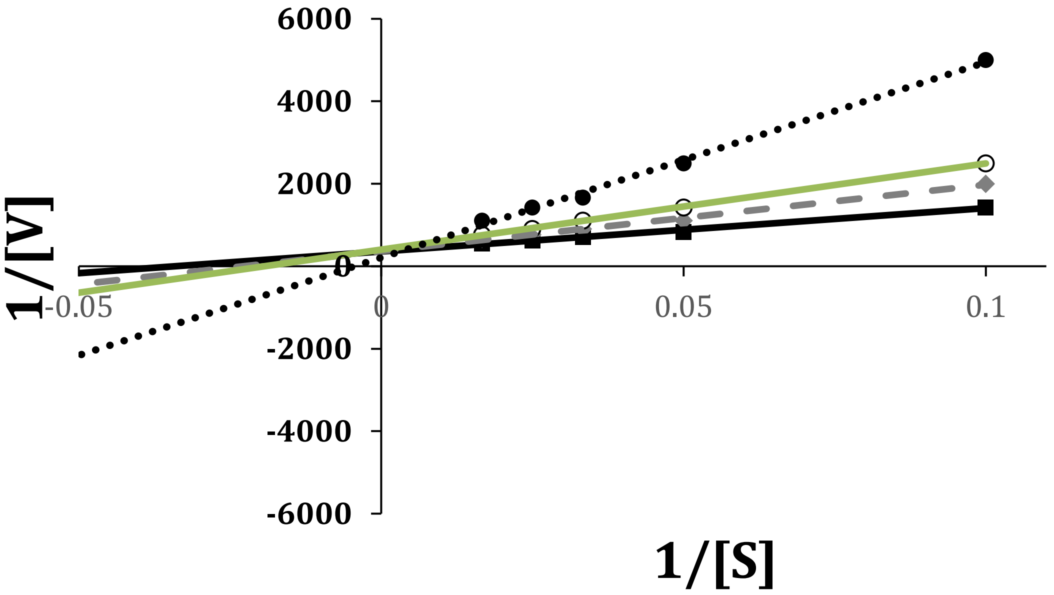

Supporting Figure S9. Qualitative analysis of LSD1 catalyzed demethylation with increasing NLS peptide concentrations indicates competitive inhibition. Lineweaver Burk plot of initial velocity measurements of ΔN LSD1-catalyzed demethylation on an H3K4me2 peptide substrates at 0 μM (squares, black line), 2.5 μM (grey diamonds, grey dashed line), 5 μM (open circles, green line), and 10 μM (solid circles, black dashed line) NLS peptide concentrations. Linear regression analysis reveals that all traces intersect the y-axis, indicative of competitive inhibition kinetics.

SFigure 10

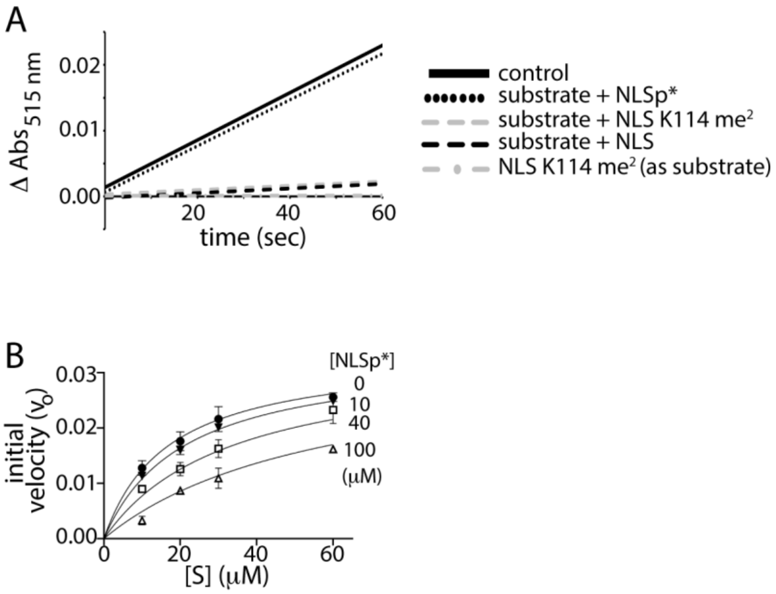

Supporting Figure S10. **Effect of methylation and phosphomimic substitutions within the NLS peptide on ΔN LSD1 catalyzed demethylation. A)** Plot of absorbance change versus time (sec) using an LSD1 - horseradish peroxidase coupled assay at 10 μM modified and unmodified NLS peptide concentrations. The NLS peptide (black dashed line) and the K114 methylated peptide (NLS K114 me2) (grey dashed line) both inhibit LSD1 activity on a H3K4me2 model peptide substrates. The NLS K114me2 peptide alone (dash-dot-dash line) also inhibits the reaction and does NOT act as a substrate of LSD1. 10 μM phosphomimic NLS peptide (14-mer NLSp*, short-black dash) does not inhibit LSD1-catalyzed demethylation on a H3K4me2 21 a.a. peptide substrate and is comparable to the control (no inhibitor, black line) experiment. **B)** For the NLSp* peptide, an approximate Ki was determined through measuring the initial velocity versus H3K4me2 21 a.a. peptide substrate concentration (**Supporting Table S2**, N=2), demonstrating a 10 fold reduction in inhibition due to the phosphomimic substitutions. All measurements were performed in an identical manner as in **Figure 4A**.

SFigure 11

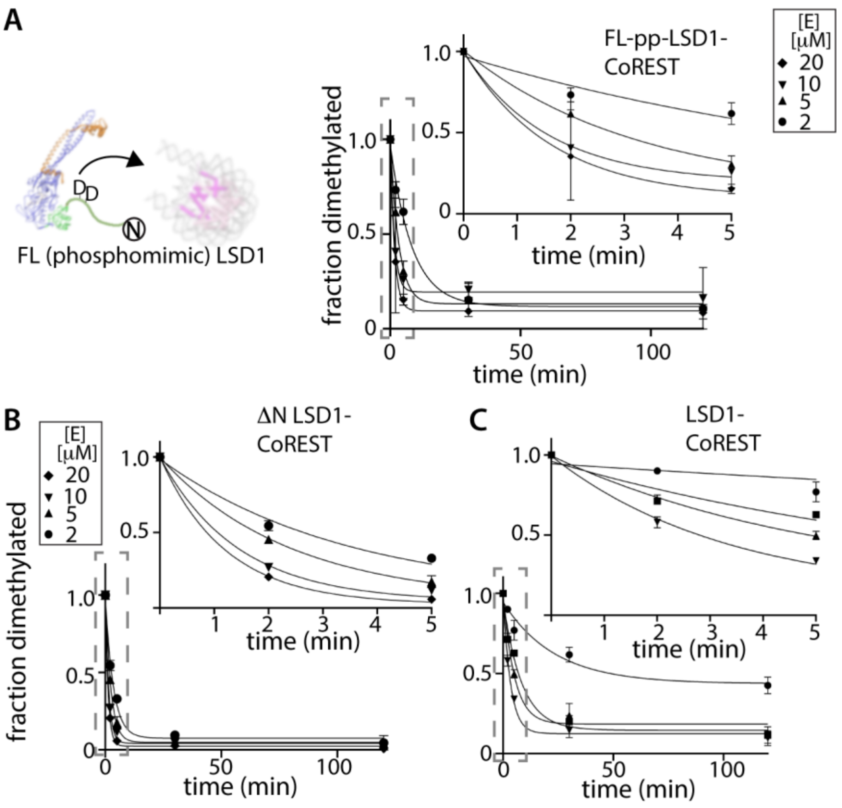

Supporting Figure S11. The phosphomimic (T110D/S111D) full length LSD1 partially restores LSD1-catalyzed demethylation on H3K4me2 nucleosome substrates. A) At left is a cartoon schematic of a (T110D/S111D) full length LSD1 engaging its nucleosome substrate. At right is a western blot image analysis based upon time course assays (**Figure 4B and Supplemental Fig S4**). This image analysis enabled a $k_{observed}$ determination for the LSD1 (T110D/S111D, aa 1-852) catalyzed reaction. Incubation of T110D/S111D-enzyme concentrations (2 (black circle), 5 (triangle), 10 (inverted triangle), and 20 μM diamond) in the buffer 50 mM HEPES (pH 8.0), 50 mM KCl, 5% glycerol, 1 mM TCEP was performed and reactions were initiated by adding 100 nM nucleosome substrate, as described in the methods. An inset panel shows the fraction of dimethylated H3K4me2 in the LSD1 (T110D/S111D, aa 1-852) sample. **Fig S11A** and **B)** allow for a clear, visual comparison of the $k_{observed}$ quantitative data acuired for **A)** (T110D/S111D) full length LSD1 (aa 1-852) purified enzyme as well as the **B)** ΔN LSD1 (171-852) and full length LSD1 (1-852) enzymes, as presented **Figure 4E/F**.

SFigure 12

A

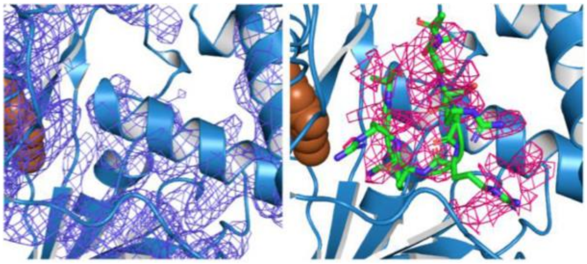

B

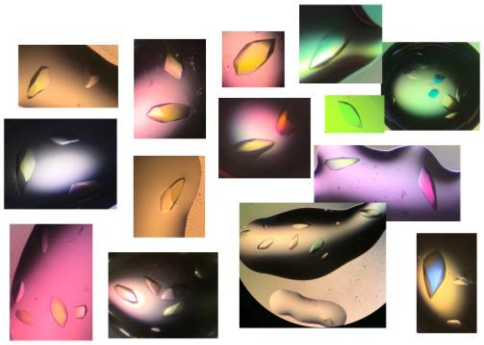

C

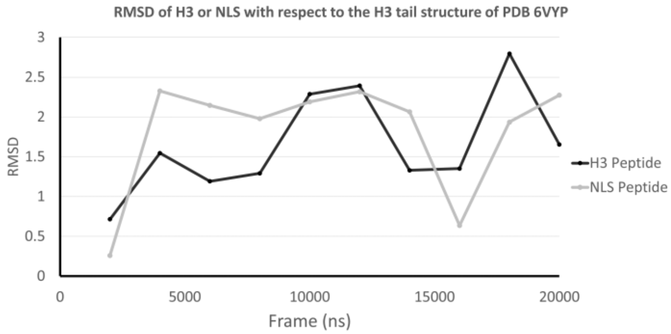

Supporting Figure S12. Combined X-ray and MD approach for comparing the H3 and NLS interaction at LSD1's active site. A) The 2Fc-Fc electron density (left, blue mesh, σ = 1.1 rmsd) and mFc-DFc map (right, pink mesh, σ = 2.3 rmsd) help identify the location of the NLS peptide (green) on the surface of LSD1 active site (blue cartoon/ribbons). This 3.5 Å low-resolution data demonstrates that transient different density (pink mesh) exists atop the LSD1 (blue cartoon) active site pocket and near FAD (brown sphere). We attribute this weak difference density to

the NLS peptide. However, the accurate placement and orientation of the NLS peptide was not possible given the low-resolution data and high anionic crystallization conditions that appear to dramatically influence the NLS-LSD1 interaction. Hence, manual modeling building of the NLS peptide (green) into the difference density was performed with guidance by the LSD1-nucleosome struture (PDB 6VYP). This model was then subject to a 20 μs AMBER based MD simulation to compare the trajectories of the NLS peptide (this work) and the H3 (PDB 6VYP) peptide within the enzyme active site cleft (**Figure 6 and methods**). **B)** Images of ΔN LSD1 (171-852)-CoREST (286-482) crystals. Three distinct and different crystallization conditions were pursued utilizing co-crystallization and crystal soaking strategies. These include condition 1: 2 M sodium formate, 0.4 M NaCl, 0.1 M citrate (pH 5.6) and 10 mM DTT, conditions 2: 0.63 M $(NH_4)_2SO_4$, 0.6 M Li_2SO_4, 0.25 M NaCl, 100 mM citrate (pH 5.6), and 5 mM TCEP), and condition 3: 1.4 M Na^+/K^+ tartrate and 100 mM N-2-acetoamido-2-iminodiacetic acid (ADA), pH 6.1. The 3.5 Å data in **A)** was collected from crystals transferred from condition 1 to a final solution of 1.8 M sodim formate, 0.1 M NaCl, 0.1 M citrate (pH 5.6), and 10 mM DTT, with 23% (v/v) glycerol, and 5 mM NLS peptide. Alternative conditions with lower anionic salt concentrations failed due to either poor diffraction or to crystals dissolving in solution. Results of this crystallographic strategy suggest that the the NLS peptide transiently interacts at the enzyme active site under crystallization conditions, consistent with an electrostatically driven interaction. Scale units for images of these specific crystals cannot be provided with accuracy, although crystals grew to their maximum size of 400 μm x 100 μm x 90 μm in approximately 14 days. **C)** Plot showing the RMSD values for 10 trajectories (2000, 4000, 6000, 8000, 10000, 12000, 14000, 16000, 18000, 20000 ns) during the course of a 20 μs MD simulation for the H3 (black, aa 3-11) or the NLS (grey, aa 110-117) peptide fragments bound to LSD1.

References:

[1] Bonneau, R., Strauss, C. E., Rohl, C. A., Chivian, D., Bradley, P., Malmstrom, L., Robertson, T., and Baker, D. (2002) De novo prediction of three-dimensional structures for major protein families, *J Mol Biol 322*, 65-78.

[2] Kim, D. E., Chivian, D., and Baker, D. (2004) Protein structure prediction and analysis using the Robetta server, *Nucleic Acids Res 32*, W526-531.

[3] Shen, Y., Lange, O., Delaglio, F., Rossi, P., Aramini, J. M., Liu, G., Eletsky, A., Wu, Y., Singarapu, K. K., Lemak, A., Ignatchenko, A., Arrowsmith, C. H., Szyperski, T., Montelione, G. T., Baker, D., and Bax, A. (2008) Consistent blind protein structure generation from NMR chemical shift data, *Proc Natl Acad Sci U S A 105*, 4685-4690.

[4] Tortorici, M., Borrello, M. T., Tardugno, M., Chiarelli, L. R., Pilotto, S., Ciossani, G., Vellore, N. A., Bailey, S. G., Cowan, J., O'Connell, M., Crabb, S. J., Packham, G., Mai, A., Baron, R., Ganesan, A., and Mattevi, A. (2013) Protein recognition by short peptide reversible inhibitors of the chromatin-modifying LSD1/CoREST lysine demethylase, *ACS Chem Biol 8*, 1677-1682.

[5] Forneris, F., Binda, C., Vanoni, M. A., Battaglioli, E., and Mattevi, A. (2005) Human histone demethylase LSD1 reads the histone code, *J Biol Chem 280*, 41360-41365.

[6] Forneris, F., Binda, C., Vanoni, M. A., Mattevi, A., and Battaglioli, E. (2005) Histone demethylation catalysed by LSD1 is a flavin-dependent oxidative process, *FEBS Lett 579*, 2203-2207.

[7] Forneris, F., Binda, C., Dall'Aglio, A., Fraaije, M. W., Battaglioli, E., and Mattevi, A. (2006) A highly specific mechanism of histone H3-K4 recognition by histone demethylase LSD1, *J Biol Chem 281*, 35289-35295.

[8] Forneris, F., Binda, C., Adamo, A., Battaglioli, E., and Mattevi, A. (2007) Structural basis of LSD1-CoREST selectivity in histone H3 recognition, *J Biol Chem 282*, 20070-20074.

[9] Culhane, J. C., Szewczuk, L. M., Liu, X., Da, G., Marmorstein, R., and Cole, P. A. (2006) A mechanism-based inactivator for histone demethylase LSD1, *J Am Chem Soc 128*, 4536-4537.
